# Women with premenstrual syndrome exhibit bodily information processing and a moderate deficit in emotional interference functioning

**DOI:** 10.3389/fpsyg.2025.1692811

**Published:** 2026-01-12

**Authors:** Yumiko Crysia Suzuki, Natsuki Saito, Hideki Ohira

**Affiliations:** Department of Cognitive and Psychological Sciences, Graduate School of Informatics, Nagoya University, Nagoya, Japan

**Keywords:** premenstrual syndrome, late luteal phase, interoceptive mismatch, emotional interference, emotional face–word Stroop task, bodily information processing, acute stress, 3-back task

## Abstract

This study examined women with premenstrual syndrome (PMS) during the late luteal phase, focusing on cognitive function under stress. Additionally, we investigated the association between cognitive performance and interoceptive processing at baseline. Acute stress was experimentally induced in women using the Trier Social Stress Test (TSST). We evaluated performance on the Emotional Face–Word Stroop task and the 3-Back task, comparing PMS and without PMS groups at pre-stress, post-stress, and recovery phases. Furthermore, we analyzed the association between baseline Emotional Interference and the Interoceptive Mismatch, which was defined as the discrepancy between interoceptive accuracy and sensibility. Mixed ANOVA revealed that, in the Emotional Face–Word Stroop task, the PMS group exhibited increased error rate without group differences in reaction time. Linear mixed-effects modeling (LMM) indicated subjective performance interference positively predicted incongruent reaction time, capturing individual variability beyond group effects. Conversely, in the 3-Back task, no group differences were observed, but LMM showed increased sensitivity from signal detection theory at post-stress and recovery phases, reflecting individual variability. Moreover, regression analysis revealed that greater Interoceptive Mismatch was significantly associated with lower Emotional Interference. This observation suggests that women with PMS exhibit a processing bias toward bodily sensations and emotions, reflecting a bottom-up style of cognitive processing. These results highlight the importance of understanding cognitive vulnerability in PMS from a multidimensional perspective, including interoception and subjective experiences. This understanding may contribute to the development of individualized support and preventive interventions for women with PMS.

## 1 Introduction

Premenstrual syndrome (PMS) refers to a cluster of psychological, physical, and behavioral symptoms that occur cyclically in association with the menstrual cycle. PMS affects approximately 50% of women of reproductive age (Hylan et al., 1999; Direkvand-Moghadam et al., 2014; Modzelewski et al., 2024) and has diverse symptoms, including abdominal pain, bloating, breast tenderness, and fatigue, as well as emotional symptoms such as depressed mood, anxiety, tension, emotional instability, and irritability—the latter being reported as one of the earliest and most severe complaints (Direkvand-Moghadam et al., 2014). Individual women typically experience similar symptom patterns across cycles, ranging from mild to severe symptoms (Bloch et al., 1997; Pearlstein et al., 2005).

The diagnostic criteria for PMS are based on the assessment of recurrent physical, psychological, and behavioral symptoms associated with the menstrual cycle. Internationally, the guidelines established by the American College of Obstetricians and Gynecologists (ACOG) and the Diagnostic and Statistical Manual of Mental Disorders, Fifth Edition (DSM-5), are widely used.

Based on these diagnostic frameworks, the Premenstrual Symptoms Screening Tool (PSST) was developed to evaluate the severity of PMS and premenstrual dysphoric disorder (PMDD) and to evaluate how they interfere with daily functioning (Steiner et al., 2003). The PSST is a self-report questionnaire and is a widely used assessment tool for identifying clinically relevant premenstrual symptoms.

PMDD is characterized by clinically significant distress and marked impairment in work, academic, social, and other areas of functioning. Moreover, PMDD is frequently associated with maladaptive behaviors, including elevated suicide risk and increased engagement in addictive behaviors (Joyce et al., 2021). These symptoms typically recur with each menstrual cycle (Henderson et al., 2025), and PMDD is regarded as an independent mood disorder in terms of treatment response and pathophysiology; it is classified as a mood disorder in the DSM-5 and as a gynecological disorder in the ICD-11.

A primary characteristic of PMS is its predictable recurrence associated with hormonal fluctuations across the menstrual cycle. Notably, although women with PMS maintain normal hormone levels (Rubinow and Schmidt, 1995), they may exhibit heightened sensitivity to the typical hormonal changes that occur throughout the cycle (Yonkers et al., 2008). Estrogen and progesterone influence the activity of brain regions involved in emotion and cognition, such as the amygdala, hippocampus, and prefrontal cortex (Del Río et al., 2018). Accordingly, the mood fluctuations and cognitive changes observed in PMS are likely to arise from interactions between these hormones and neural networks (Fruzzetti and Fidecicchi, 2020). Furthermore, the effects of these hormones display phase-specific characteristics that vary across the menstrual cycle and may be amplified by rapid hormonal shifts or exposure to stress (Rubinow and Schmidt, 1995; Bloch et al., 1997).

When PMS and PMDD are conceptualized along a continuum, PMDD is placed on the most severe end (Naik et al., 2023; Modzelewski et al., 2024). However, because these diagnoses rely heavily on subjective symptom ratings, accurate identification remains challenging. Furthermore, many women do not seek medical consultation for their symptoms, leading to delayed diagnosis or underdiagnosis (del Mar Fernández et al., 2019).

The current study is based on the assumption that women with PMS may have difficulties processing internal bodily states, such as visceral sensations and hormonal fluctuations. Interoception—the perception of internal bodily signals—is essential for emotion generation and the maintenance of self-regulation (Khalsa et al., 2018). Garfinkel et al. (2015) demonstrated that interoception consists of three dimensions—accuracy, sensibility, and awareness—each representing an independent construct. Furthermore, Nord and Garfinkel (2022) noted that interoceptive processing is shaped by multilayered mechanisms. They highlighted that discrepancies between top-down beliefs or predictions and afferent bodily signals may play crucial roles in understanding diverse neuropsychiatric conditions.

From this perspective, *the interoceptive trait prediction error* (ITPE) proposed by Garfinkel et al. (2015) is an index that quantitatively captures the discrepancy between interoceptive accuracy and interoceptive sensibility. Previous studies have indicated that this discrepancy reflects physiological and cognitive vulnerabilities associated with difficulties in emotion regulation and may function as a transdiagnostic marker operating across diagnostic categories (Garfinkel et al., 2016; Nord and Garfinkel, 2022). Based on this theoretical framework, herein, we defined the difference between interoceptive accuracy and interoceptive sensibility as an *interoceptive mismatch*.

We previously reported that women with PMS exhibit a mismatch characterized by high interoceptive accuracy and low subjective awareness (Suzuki and Ohira, 2025). Thus, bodily signals may influence emotional experience without being sufficiently brought into conscious awareness. Importantly, the term *interoceptive awareness* used in our previous study conceptually corresponds to *interoceptive sensibility* in the three-dimensional model proposed by Garfinkel et al. (2015). Taken together, the interoceptive mismatch observed in women with PMS may act maladaptively within mental-health contexts and likely contributes to emotional instability and difficulties in self-regulation, functioning as a form of transdiagnostic vulnerability (Garfinkel et al., 2016).

Women with PMS also show heightened vulnerability to stress. This vulnerability manifests as increased negative affective responses (Liu et al., 2017; Suzuki and Ohira, 2025), enhanced attentional bias toward negative stimuli (Eggert et al., 2016, 2017), and atypical autonomic nervous system (ANS) regulation following stress (Liu et al., 2018; Suzuki and Ohira, 2025). Several studies have further suggested that women with PMS exhibit impairments in executive functions such as inhibitory control (e.g., bodily sensations or facial expressions), conflict monitoring and resolution under semantic interference), working memory, and cognitive flexibility; however, findings are inconsistent (Hoyer et al., 2013; Eggert et al., 2017; Slyepchenko et al., 2017; Aoki et al., 2022). Moreover, studies on PMDD have reported more pronounced working-memory deficits (Yen et al., 2012); such inconsistencies may reflect differences in hormonal sensitivity or individual variations in stress vulnerability (Henderson et al., 2025).

According to Pessoa (2009), under high emotional arousal, cognitive resources may shift toward emotion processing at the expense of executive control—a phenomenon called “executive competition.” This effect may be particularly pronounced in women with PMS during the late luteal phase when stress is heightened (Hoyer et al., 2013). Based on these findings, we considered that experimentally inducing acute stress—which increases emotional load and restricts cognitive resources—would be an effective method for revealing cognitive characteristics associated with PMS. Therefore, the first aim of this study was to examine cognitive functioning in women with PMS during the late luteal phase and to clarify how stress influences performance on cognitive tasks.

To evaluate both emotion-laden cognitive processes (hot cognition) and emotion-independent cognitive processes (cold cognition), we employed tasks corresponding to each domain. In addition, to control for variations in arousal levels, all tasks were administered from late morning to early afternoon. Because women with PMS exhibit delayed recovery from stress (Suzuki and Ohira, 2025), we predicted that group differences in cognitive performance would become more evident during the recovery phase. Furthermore, we hypothesized that cognitive performance under stress in the PMS group would be predicted by subjective stress intensity (Shields et al., 2016) and subjective appraisals such as task confidence and perceived performance interference (Robison and Unsworth, 2017).

Meanwhile, previous studies have shown that interoceptive mismatch is associated with difficulties in emotion regulation (Nord and Garfinkel, 2022). Therefore, we hypothesized that, among women with PMS, increased emotional interference under stress would be predicted by larger interoceptive mismatch as a stable individual trait. Thus, the second aim of this study was to determine how interoceptive mismatch predicts emotional interference scores across pre-stress, post-stress, and recovery phases.

In summary, this study aimed to elucidate how PMS influences the interplay between cognitive performance, emotion regulation, and interoceptive processing under stress. By integrating cognitive tasks with the interoceptive mismatch index, we sought to obtain a comprehensive understanding of the mechanisms underlying the difficulties encountered by women with PMS in their daily lives.

## 2 Materials and methods

### 2.1 Participants

The study was approved by the university's ethics committee for psychological research and conducted in accordance with the Declaration of Helsinki.

The participant cohort in this study was the same as that reported in our previous study (Suzuki and Ohira, 2025). Recruitment was conducted through the university's internal participant-registration platform. A total of 90 students affiliated with the same University, comprising undergraduate, graduate, and non-degree students, participated in the study. Data from two participants (one Japanese and one international) were excluded owing to withdrawal from the study. Exclusion criteria included the use of oral contraceptives, pregnancy or lactation, current psychiatric treatment, use of medications that may affect stress responses, or markedly irregular menstrual cycles.

All participants were women and were classified into either the PMS group (*n* = 21) or without PMS group (*n* = 67) based on strict criteria using PSST scores, self-reported menstrual-cycle tracking over two consecutive cycles, and an initial screening evaluation (PSST). However, because the PSST is a self-report instrument and does not constitute a clinical diagnosis, the possibility of misclassification should be acknowledged.

In addition, five individuals who met the PMDD score threshold were included in the PMS group in accordance with prior research conceptualizing PMDD as a severe subtype within the PMS spectrum (Naik et al., 2023; Modzelewski et al., 2024). Although the group sizes were unequal, this imbalance reflected the prevalence and recruitment feasibility of PMS rather than sampling error. Therefore, statistical analyses controlled for differences in group size. A sensitivity analysis using G^*^Power 3.1 indicated that, with α = 0.05 and power = 0.80, the minimum detectable effect size was *d* = 0.63 (Faul et al., 2009), corresponding to a medium-to-large effect size.

Participants (aged 18–34 years) recorded the onset and end dates of their menstrual cycles across two consecutive cycles. Based on the two-cycle menstrual tracking, the researchers estimated the timing of each participant's next ovulation. A pharmacist instructed participants on how to use an ovulation test kit (Do-test LH II, Rohto Pharmaceutical Co.), and participants identified their luteinizing hormone surge using the kit. The researchers then estimated the projected onset of the next menstruation based on these results.

Laboratory sessions were conducted 1–7 days before the estimated onset of the next menstruation. After the experiment, the actual onset of menstruation was confirmed by participant report, and the timing of the session was retrospectively verified to have occurred during the late luteal phase.

### 2.2 Cognitive function assessment

#### 2.2.1 Emotional face–word stroop task

To evaluate cognitive control under emotional interference, we employed the Emotional Face–Word Stroop task, which measures the conflict arising when emotional facial expressions and overlaid emotional words are incongruent (Stroop, 1992; Etkin et al., 2006). In incongruent trials, participants must inhibit interference from the semantic content of the word and respond based solely on the facial expressions, thereby engaging semantic inhibitory control and attentional processes (Etkin et al., 2006; Ovaysikia et al., 2011).

Stimuli were selected from the National Institute of Advanced Industrial Science and Technology Facial Expression Database, comprising facial expressions (happy or anger) presented by eight models (four male and four female). Each face was overlaid with the words “happy” or “angry” in red font. Trials were either congruent (for example, happy face + “happy”) or incongruent (for example, happy face + “angry”) (Fujimura and Umemura, 2018).

**Figure 1:**
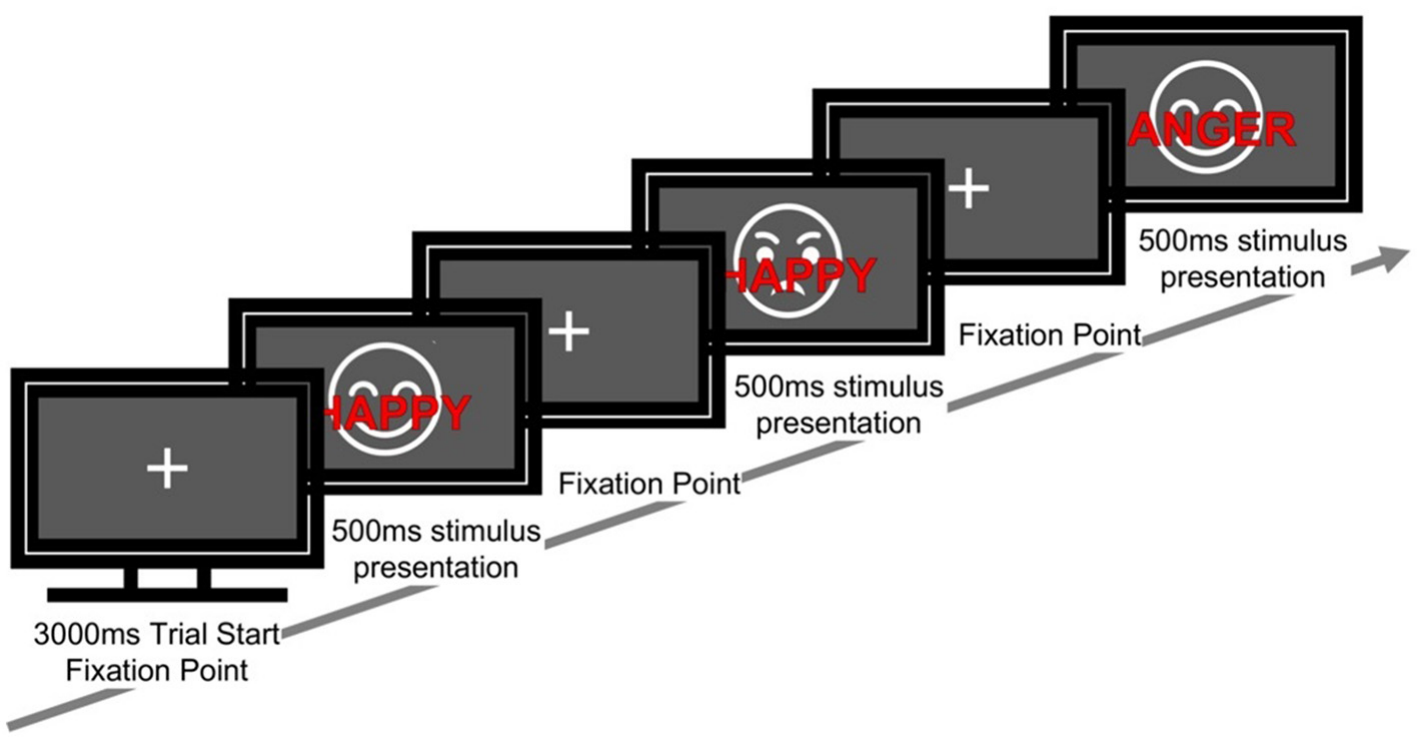
Sequence of a single trial in the emotional face–word stroop task. Each trial started with a fixation cross (3,000 ms), followed by a face with an overlaid emotional word (500 ms). The word was either congruent or incongruent with the facial expression. Participants responded to the emotion of the face while ignoring the words.

The task comprised 120 trials in total. Each trial proceeded as follows: a fixation cross for 3,000 ms, followed by a stimulus for 500 ms, and a response window of 2,500 ms. Participants were instructed to ignore the meaning of the word and to identify the facial expression as quickly and accurately as possible (Figure 1).

Participants were instructed to respond by pressing the “F” key with the left index finger and the “J” key with the right index finger. The mapping between facial expression category (happy/angry) and response key (F/J) was counterbalanced across participants (for example, bodily sensations or facial expressions) half of the participants were assigned happy → F and angry → J, whereas the other half were assigned happy → J and angry → F). The order of stimulus presentation was pseudorandomized. Before beginning the task in Phase 1 (P1), participants completed 12 practice trials with feedback to ensure comprehension of the task.

The stimulus presentation ratios were as follows: happy and angry facial expressions were each presented with a probability of 50%. Congruent to incongruent trials were presented at a ratio of 70%−30%, respectively. Furthermore, although the task was presented as a continuous sequence, it was divided into two analytic blocks (first half and second half). Within each block, the proportions of congruent to incongruent trials (70%:30%) and happy to angry expressions (50%:50%) were maintained. The total number of stimuli for each condition was also matched across the first and second halves of the task.

Reaction time (RT) and error rates were calculated under the following conditions:

- Congruency Condition: congruent (C) vs. incongruent (IC)
- Sequential Context: based on the congruency of the preceding trial, each trial was categorized into one of four types: C_C, IC_C, C_IC, or IC_IC
- Emotion Condition: happy (HA) vs. angry (AN). For each emotion, congruent and incongruent conditions were labeled as: HA_C, HA_IC, AN_C, and AN_IC
- Trial Half: C and IC trials were analyzed separately for the First Half (FH) and Second Half (SH) of the task.
- Emotional Interference: calculated as the RT difference between incongruent and congruent trials (Diff_IC–C_RT)
- Attentional bias: calculated as the RT difference between angry and happy faces (Diff_AN–HA_RT)

#### 2.2.2 3-Back task

To evaluate working memory and executive function, we employed a 3-Back task that assesses updating memory, inhibitory control, and attentional shifting (Kane et al., 2007). Participants were required to determine whether the currently presented digit matched the one shown three trials earlier.

The task consisted of 120 trials, with the first three trials excluded from all analyses. Stimuli were white digits (0–9) presented on a black background. The order of stimulus presentation was pseudorandomized, and the frequency of occurrence for each digit was balanced across the task. Furthermore, although the task was presented as a continuous sequence, it was divided into two analytic halves—the first half (FH; Trials 5–62) and second half (SH; Trials 63–120). Within these halves, the numbers of target and non-target trials, as well as the total number of stimuli in each category, were matched across the two segments.

The sequence of events for each trial was as follows: a fixation cross was presented for 3,000 ms, followed by the stimulus for 500 ms, after which participants were given 2,500 ms to respond.

**Figure 2:**
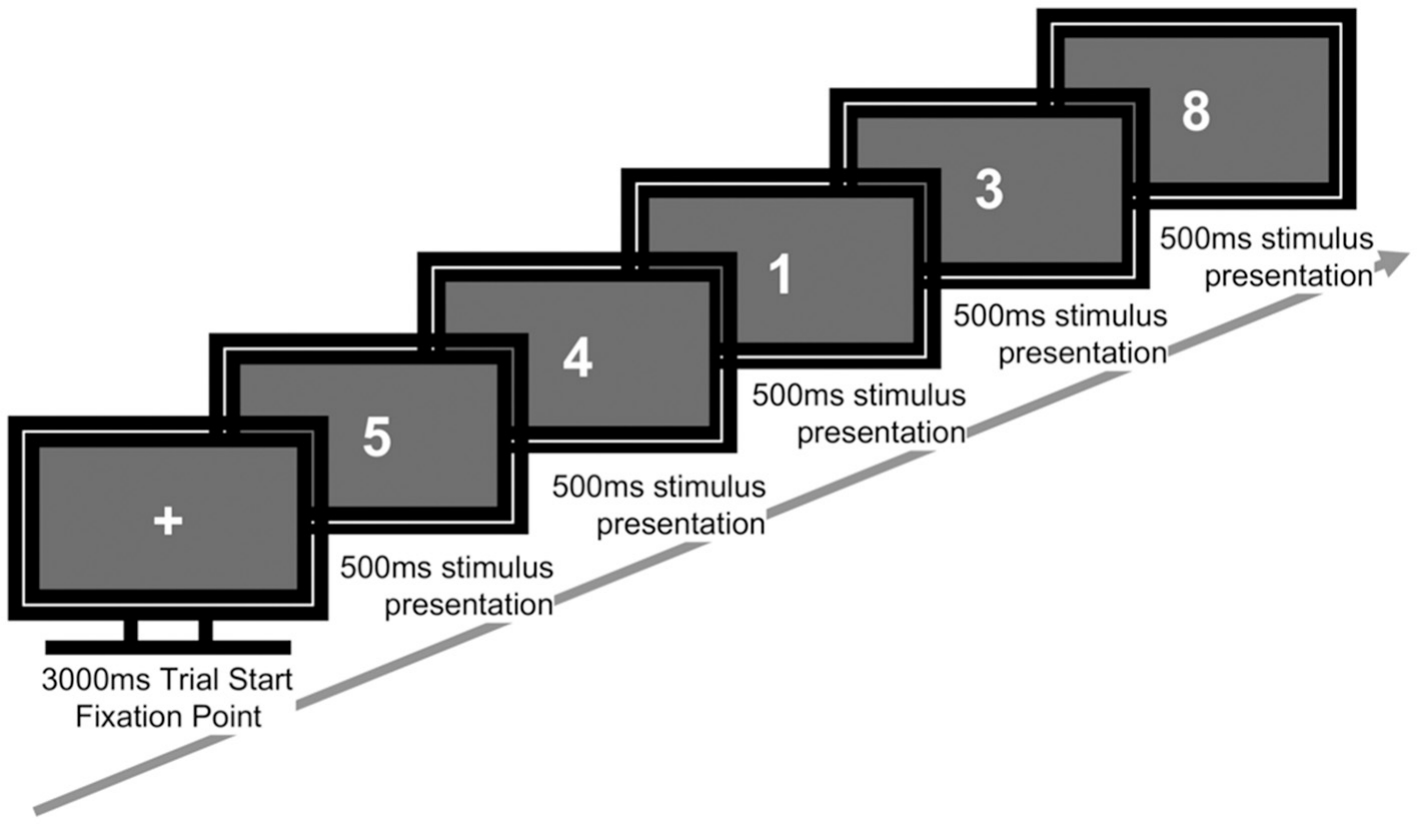
Sequence of a single trial in the 3-back task. Each trial began with a fixation cross (3,000 ms), followed by a sequence of numbers presented individually (500 ms each). Participants responded whether the current number matched the one shown three trials earlier.

Participants were instructed to make a “target” response when the current stimulus matched the digit presented three trials earlier (i.e., a 3-back target), and to make a “non-target” response otherwise. For target responses, participants pressed the “J” key with the right index finger; for non-target responses, they pressed the “K” key with the right middle finger. The key–response mapping was fixed for all participants (Figure 2). No feedback was provided during the task. Before beginning the task in Phase 1 (P1), participants completed 12 practice trials with feedback to ensure comprehension of the task.

The experimental conditions were as follows:

- Stimulus ratios: target trials (in which the digit matched that from three trials earlier) accounted for 33.33%, and non-target trials accounted for 66.67% of all trials.
- Experimental conditions

- Hit (H): a correct response to a target trial
- Miss (M): an incorrect rejection of a target trial
- Correct Rejection (CR): a correct response to a non-target trial
- False Alarm (FA): an incorrect response identifying a non-target trial as a target
- Empty: no response was made within the response window

- Dependent measures

- Hit rate = H/(H + M)
- False Alarm rate = FA/(FA + CR)
- Correct RT: the average RT for correct responses (H and CR)
- Accuracy = (H + CR)/Total Trials (excluding Empty trials)
- Correct RT_FH, Correct RT_SH, Accuracy_FH, Accuracy_SH
- Empty rate: proportion of trials with no response

- Signal detection theory (SDT) Indices (Snodgrass and Corwin, 1988)

- Sensitivity index (dL): dL = ln[(H × (1 – FA))/((1 – H) × FA)]
- Criterion level (CL): CL = 0.5 × ln[((1 – FA) × (1 – H))/(H × FA)]

#### 2.2.3 Experimental environment

The Emotional Face–Word Stroop and 3-Back tasks were implemented on a Windows PC using MATLAB 2019b (MathWorks Inc.) and Psychtoolbox-3. RTs were recorded in milliseconds.

### 2.3 Interoceptive mismatch index

The Interoceptive Mismatch Index was computed as an indicator of the discrepancy between interoceptive accuracy and interoceptive sensibility, based on the framework proposed by Garfinkel et al. (2015). In this study, interoceptive accuracy was assessed using the heartbeat counting task (Schandry, 1981; Garfinkel et al., 2015). In this task, participants were instructed to silently count the number of heartbeats they “truly felt” without palpating their pulse. The task consisted of three trials of 25, 35, and 45 s, during which actual heartbeats were recorded using a BIOPAC system (ECG100C) at a sampling rate of 1,000 Hz. Interoceptive accuracy was indexed by the Heartbeat Counting Task (HCT) score, calculated using the following formula:


(1) $$\begin{array}{l} 1/3\;\Sigma \;(1|recorded\;heartbeatscounted\;heartbeats|\\ \;/recorded\;heartbeats). \end{array}$$

Interoceptive sensibility was measured using the Japanese version of the Multidimensional Assessment of Interoceptive Awareness (MAIA; Mehling et al., 2012). Both interoceptive accuracy and interoceptive sensibility scores were standardized (z-transformed) across the entire sample (*n* = 88), with a mean of 0 and a standard deviation of 1.

Thereafter, the Interoceptive Mismatch Index was calculated by subtracting the z score of interoceptive sensibility from the z score of interoceptive accuracy. This index reflects a simple difference score (accuracy z – sensibility z) obtained after independently standardizing the two measures, without applying any differential weighting. Thus, the two indices were treated as contributing equally. Higher values on the Interoceptive Mismatch Index indicate a state in which objective accuracy of bodily signal perception is relatively greater than subjective bodily sensibility.

Z-standardization was performed using the following formula:


$$\begin{array}{l} z\;=\frac{x-mean\;x}{SD} \end{array}$$ (2)

In this study, the two measures (interoceptive accuracy and interoceptive sensibility) were z-standardized independently prior to computing their difference. Consequently, neither measure was differentially weighted, and both were treated as equally important contributors. A higher mismatch index score therefore reflects a relatively greater dominance of interoceptive accuracy over subjective interoceptive sensibility.

### 2.4 Procedures

#### 2.4.1 PMS screening

PMS screening was conducted using the PSST developed by Steiner et al. (2003). The Japanese version of the PSST, whose validity and reliability were confirmed by Miyaoka (2009), was used in this study. The PMS group in this study refers to women reporting PMS symptoms on the PSST and does not refer to clinically diagnosed PMS. The PSST has been widely used in non-clinical populations, including university students, to classify PMS symptom severity in research settings (Mirghafourvand et al., 2015; Slyepchenko et al., 2017).

Participants were asked to indicate whether they had experienced any symptoms listed in the PSST that started before their period and stopped within a few days of bleeding. Participants with possible moderate-to-severe PMS or PMDD were defined using the following criteria:

- Rated at least one of the following symptoms as “moderate” or “severe”: anger/irritability, anxiety/tension, tearfulness/increased sensitivity to rejection, or depressed mood/hopelessness.
- Endorsed at least four of the 12 premenstrual symptom items as “moderate” or “severe.”
- Endorsed at least one item in the domain of impairment in work or school, social activities, or relationships as “moderate” or “severe.”

Participants who met these criteria were classified as women with PMS, whereas all others were classified as women without PMS (“none-to-mild PMS”). The score derived from the PSST item “Interfered with your work efficiency or productivity” was defined as the Subjective Performance Interference Score (SPI). The SPI score distribution is presented in Supplementary material S1

#### 2.4.2 Experimental procedures

Participants were instructed to avoid vigorous exercise on the day prior to the experiment, obtain sufficient sleep, maintain regular meals, and refrain from alcohol consumption. The laboratory environment was kept quiet and well ventilated. Upon arrival, participants first performed a mouth rinse, followed by a body temperature measurement. After providing informed consent, a portable heart rate monitor (myBeat WHS-1, UNION TOOL CO., Japan) was attached to the left subclavicular region to record heart rate continuously throughout the experiment.

All experimental sessions began 10:30 in the morning. Although start times varied by approximately ±30 min across participants, the phase structure and timing of all measurements were strictly standardized across individuals. This scheduling was chosen to minimize the influence of circadian fluctuations on cognitive performance (Schmidt et al., 2007).

The experiment consisted of three main phases: pre-stress (P1), post-stress (P2), and recovery (P3). Acute stress was induced between P1 and P2 using the Trier Social Stress Test (TSST; Kirschbaum et al., 1993) A 30-min waiting period (WT) was inserted between P2 and P3. During each phase (P1, P2, and P3), participants completed cognitive tasks, responded to questionnaires, and provided saliva samples. Questionnaires and saliva samples were also collected during WT.

**Figure 3:**
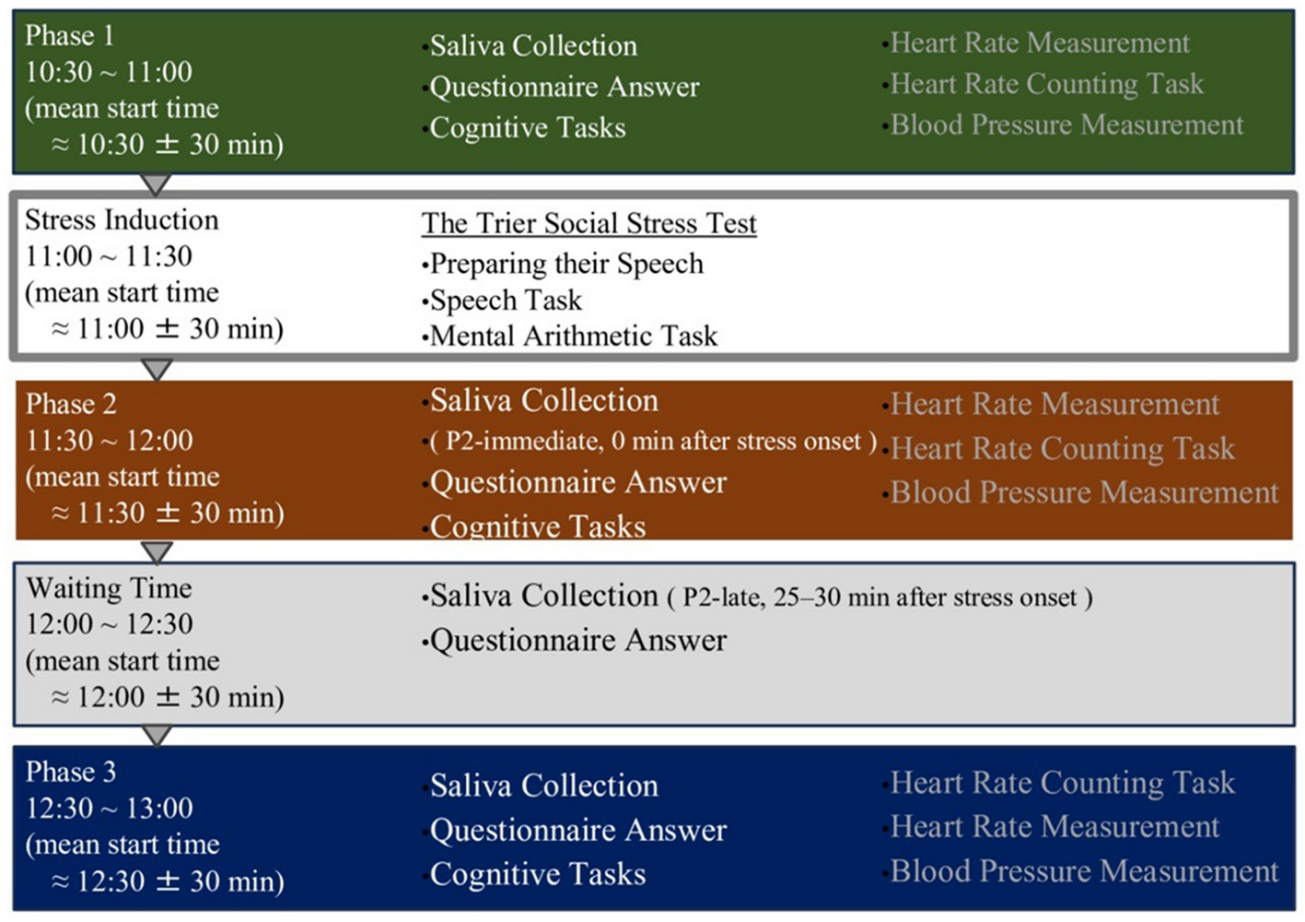
Overview of the experimental timeline and procedures. The experiment consisted of four phases: a baseline phase (Phase 1), stress induction phase using the Trier Social Stress Test (TSST), post-stress phase (Phase 2), and recovery phase (Phase 3). Saliva samples, questionnaires, cognitive tasks, and physiological measurements were performed in all phases. Saliva was collected immediately and 25–30 min after stress onset to assess hormonal responses.

Heart rate was continuously recorded for 7 min during each phase using a BIOPAC MP150 system with an ECG100C module, and blood pressure was measured once during each phase. After completing all procedures, the portable heart rate monitor was removed, and participants were debriefed by the researcher. The total duration of the experimental session was approximately 3 h (Figure 3).

#### 2.4.3 Stress induction

Stress was induced using TSST, with all instructions provided in Japanese (Kirschbaum et al., 1993). The TSST protocol comprised two main phases: preparation and task execution, during which participants performed speech and mental arithmetic tasks. These two tasks were designed to elicit psychological and physiological stress responses.

In the preparation phase, participants were taken to a room and briefed on the task. They were asked to give a good description of themselves and given 5 min to prepare.

The task implementation phase consisted of a speech task and a mental arithmetic task.

During the 5-min speech task, participants delivered a prepared speech in front of an evaluator (the experimenter) dressed in a white lab coat. The evaluator maintained a neutral or slightly critical facial expression, and participants were informed that their speech would be recorded and evaluated. If participants finished their speech early, they were instructed to continue speaking on the same topic.

Following the speech task, a 5-min mental arithmetic task was administered. Participants were instructed to repeatedly subtract 13 from a given number (for example, bodily sensations or facial expressions 2,097) as quickly and accurately as possible. When an error occurred, they were told to restart from the initial number.

#### 2.4.4 Subjective stress scores

Subjective stress levels were assessed using an 8-cm horizontal Visual Analog Scale (VAS), with all instructions presented in Japanese. Participants were asked to indicate their current stress levels by placing a mark along the line, where 0 (left endpoint) indicated “no stress at all” and 8 (right endpoint) indicated “extreme stress.” The results of this measure were previously reported by Suzuki and Ohira (2025) and were included as one of the fixed effects in the current study's linear mixed-effects model (LMM) analyses.

### 2.5 Data analysis

To evaluate group differences at baseline (P1), independent sample *t*-tests (Welch's *t*-test, assuming unequal variances) were conducted on cognitive task performance (Emotional Face–Word Stroop and 3-Back tasks) and cortisol concentration.

To assess the effects of temporal changes in stress, mixed-design analyses of variance (ANOVAs) were performed. For cognitive task indices, group (PMS vs. without PMS) was treated as a between-subject factor, and Time [pre-stress baseline (P1), post-stress (P2), and recovery (P3)] as a within-subject factor. *Post-hoc* comparisons using Holm correction were conducted as necessary (for example, P1 vs. P2, P1 vs. P3, P2 vs. P3 or P1 vs. P2_immediate, P1 vs. P2_late, P1 vs. P3, P2_immediate vs. P2_late, P2_immediate vs. P3, and P2_late vs. P3). Details of cortisol processing and additional *post-hoc* comparisons are provided in the Supplementary material S2.

Sphericity violations were evaluated using Mauchly's test. When sphericity was violated, the degrees of freedom (*df* 1 and *df* 2) for the corresponding within-subject effects (for example, Group, Group × Time) were adjusted using the Greenhouse–Geisser epsilon (ε) obtained from Mauchly's test. The *F*-values reported for these effects are based on the adjusted degrees of freedom.

To identify cognitive performance predictors, LMMs were conducted with Group (PMS vs. without PMS) and Time (P1–P3) as fixed effects. The models included random intercepts for participants and fixed effects for subjective stress scores, and SPI scores (task interference index based on PSST), as well as their interactions (Group × Time × Subjective Stress). The models were estimated using restricted maximum likelihood (REML). To obtain robust inference for fixed effects, cluster bootstrap (1,000 replicates; clustered by participant) was used to compute percentile-based 95% confidence intervals. Dependent variables in the Emotional Face–Word Stroop task included the reaction time under incongruent conditions (IC_RT), the emotional interference index (Diff_IC–C_RT), and attentional bias (Diff_AN–HA_RT). For the 3-Back task, sensitivity (*dL*) and decision criterion (*CL*) based on SDT were used as dependent variables. All models were estimated using restricted maximum likelihood, with PMS group and P1 set as the reference levels in dummy coding.

To examine the relationship between interoceptive mismatch and emotional interference, a simple regression analysis was conducted with emotional interference at baseline (P1) (Diff_IC–C_RT) as the dependent variable and the Interoceptive Mismatch Index as the predictor. Pearson's correlation coefficients were also computed.

All analyses were performed using HAD v18 (Shimizu, 2016) and Python. The significance level was set at *p* < 0.05. Significant results were marked with ^*^, ^**^, and ^***^ (*p* < 0.05, 0.01, and 0.001, respectively), and non-significant results were indicated as “*n.s*.” Effect sizes are reported as Cohen's *d*, partial η^2^, standardized regression coefficient (β), and *R*^2^.

## 3 Results

### 3.1 Participant characteristics

The mean participant age was 20.568 ± 2.577 years (PMS group: 21.250 ± 3.905 years; without PMS group: 20.268 ± 2.029 years), and the mean age at menarche was 12.013 ± 1.354 years (PMS group: 11.842 ± 2.007 years; without PMS group: 12.085 ± 1.083 years).

### 3.2 Pre-stress

Independent *t*-tests revealed no significant differences between the PMS and without PMS groups at baseline (P1) in Emotional Face–Word Stroop task performance, or 3-Back task performance, and cortisol concentration. However, the PMS group [*M* = 3.100, *SD* = 0.641, 95% *CI* (2.815, 3.385)] showed significantly higher SPI scores than the without PMS group [*M* = 1.485, *SD* = 0.532, 95% *CI* (1.357, 1.614)], *t* (27.180) = 10.275, *p* < 0.001, Cohen's *d* = 2.868 (Supplementary material S3).

### 3.3 Effects of stress: pre-stress, post-stress, and recovery

To assess the effects of stress, cognitive task performance was evaluated at three time points (P1, P2, and P3) using mixed-design ANOVAs.

#### 3.3.1 Emotional face–word stroop task

A main effect of Group was observed for the error rate in the second half of the task, with the PMS group exhibiting a higher error rate than the without PMS group (*p* = 0.034).

A main effect of Time was also noted. Reaction times decreased progressively from P1 to P3 (C_RT, IC_RT, C-C_RT, IC-C_RT, IC-IC_RT, HA_RT, HA_C_RT, HA_IC_RT, AN_RT, AN_C_RT, C_RT_FH, IC_RT_FH, C_RT_SH, Diff_AN-HA_RT; all *ps* < 0.05, with several effects reaching *p* < 0.01 or *p* < 0.001).

In contrast, error rates increased from P1 to P3 (C_Error, C-C_Error, IC_Error, C-IC_Error, IC-IC_Error, HA_Error, HA_C_Error, AN_Error, AN_IC_Error, Error_FH; all *ps* < 0.05, with several effects reaching *p* < 0.01).

However, the Group × Time interaction was not significant for any of the indicators (*ps* > 0.05) (Supplementary material S4).

#### 3.3.2 3-Back task

Neither the main effect of Group nor the Group × Time interaction was significant for any of the indicators (all *ps* > 0.05).

In contrast, a main effect of Time was observed across multiple indicators. From P1 to P3, Hit rate and Accuracy increased, whereas False Alarm rate, Correct RT, Correct RT_FH, and Correct RT_SH decreased. In addition, the sensitivity index *dL* increased over time. All of these effects were statistically significant (all *ps* < 0.01, with several effects reaching *p* < 0.001) (Supplementary materials S4).

### 3.4 Mixed linear model regression results

Mixed linear effects models were employed to examine the effects of Group (PMS vs. without PMS), Time (P1–P3), and individual-level predictors (including subjective stress and SPI score) on cognitive task performance. Only results with *p-*values less than 0.05 in the LMM and bootstrap-derived confidence intervals that did not include zero were reported.

#### 3.4.1 Emotional face–word stroop task

The SPI score, indicating subjective interference with task performance, was significantly positively associated with IC_RT (β = 28.925, *p* = 0.040, LMM-derived 95% *CI*: [1.342, 56.507], and bootstrap-derived 95% *CI*: [1.922, 50.301]. No other predictors reached statistical significance (Table 1).

**Table 1:** Results of the linear mixed-effects model (LMM) for the emotional face–word stroop task.

**Emotional stroop task**			**LMM results**						**Bootstrap results**			
**Indices**	**Predictor**		β **(Coef.)**	***SE***	***z***	***p*** **-value**	***CI*** **low**	***CI*** **high**	**Base_estimate**	**Boot_mean**	***CI*** **low**	***CI*** **high**
IC_RT	Intercept		647.549	13.149	49.248	0.000	621.777	673.320	-	-	-	-
	Group		−38.467	36.994	−1.040	0.298	−110.975	34.041	−38.467	−33.438	−89.045	26.824
	Time	P2	−12.519	10.608	−1.180	0.238	−33.311	8.274	−12.519	−12.260	−24.090	1.529
		P3	−24.298	10.624	−2.287	0.022	−45.121	−3.474	−24.298	−24.673	−46.798	4.529
	Subj. Stress		−3.856	9.842	−0.392	0.695	−23.147	15.435	−3.856	−4.774	−18.396	8.135
	Group × time × subj. stress	P2	−3.341	25.882	−0.129	0.897	−54.070	47.387	−3.341	−6.203	−52.219	30.640
		P3	−18.585	25.009	−0.743	0.457	−67.603	30.432	−18.585	−19.964	−65.565	18.923
	SPI score		28.925	14.073	2.055	0.040	1.342	56.507	28.925	27.579	1.922	50.301
Diff_IC-C_RT	Intercept		66.318	7.520	8.819	0.000	51.579	81.058	-	-	-	-
	Group		−24.564	20.411	−1.203	0.229	−64.571	15.442	−24.564	−21.554	−53.650	18.033
	Time	P2	−6.397	8.733	−0.733	0.464	−23.513	10.719	−6.397	−6.171	−14.963	3.156
		P3	−1.240	8.730	−0.142	0.887	−18.352	15.872	−1.240	−1.369	−17.593	23.204
	Subj. stress		2.385	7.340	0.325	0.745	−12.001	16.771	2.385	2.135	−6.202	11.227
	Group × time × subj. stress	P2	−6.192	20.336	−0.305	0.761	−46.052	33.667	−6.192	−6.149	−28.946	18.927
		P3	−7.232	19.802	−0.365	0.715	−46.045	31.580	−7.232	−8.634	−31.545	17.334
	SPI score		11.039	6.701	1.647	0.099	−2.095	24.173	11.039	10.275	−7.872	25.764
Diff_AN-HA_RT	Intercept		0.982	6.305	0.156	0.876	−11.376	13.339	-	-	-	-
	Group		3.859	17.434	0.221	0.825	−30.312	38.029	3.859	4.198	−30.835	38.081
	Time	P2	−10.265	6.423	−1.598	0.110	−22.853	2.324	−10.265	−10.459	−19.565	−2.385
		P3	−3.573	6.435	−0.555	0.579	−16.186	9.040	−3.573	−3.615	−19.723	8.088
	Subj. stress		−1.586	5.668	−0.280	0.780	−12.694	9.523	−1.586	−1.129	−8.310	6.110
	Group × time × subj. stress	P2	0.212	15.324	0.014	0.989	−29.823	30.246	0.212	2.951	−18.027	31.796
		P3	2.848	14.874	0.191	0.848	−26.305	32.001	2.848	4.425	−20.266	30.522
	SPI score		−3.079	6.151	−0.501	0.617	−15.136	8.977	−3.079	−2.893	−15.222	12.001

No significant effects of group, time, subjective stress, or their interactions were observed for any of the indices. However, higher SPI scores significantly predicted slower incongruent reaction times (IC_RT; β = 28.93, p = 0.040; bootstrap 95% CI [1.92, 50.30]), suggesting that greater subjective productivity interference was associated with reduced performance efficiency. By contrast, neither the emotional interference score (Diff_IC–C_RT) nor the attentional bias score (Diff_AN–HA_RT) showed significant effects.

Diff_AN–HA_RT, Attentional Bias Score (Angry – Happy RT); Diff_IC–C_RT, Emotional Interference Score (Incongruent – Congruent RT); IC_RT, Incongruent Reaction Time; SPI, Subjective Productivity Interference; Subj. Stress, Subjective Stress level.

#### 3.4.2 3-Back task

A significant main effect of Time was observed for the dL, with higher dL scores at both the post-stress phase (P2: β = 1.426, *p* < 0.001, LMM-derived 95% *CI*: [1.044,1.808], and bootstrap-derived 95% *CI*: [1.073, 1.793]) and the recovery phase (P3: β = 1.843, *p* < 0.001, LMM-derived 95% *CI*: [1.456, 2.229], and bootstrap-derived 95% *CI*: [1.444, 2.218]). No other predictors were significant (Table 2).

**Table 2:** Results of the linear mixed-effects model (LMM) for the 3-back task.

**3-Back task**			**LMM results**						**Bootstrap results**			
**Indices**	**Predictor**		β **(Coef.)**	***SE***	***z***	***p*** **-value**	***CI*** **low**	***CI*** **high**	**base_estimate**	**boot_mean**	***CI*** **low**	***CI*** **high**
*dL*	Intercept		4.408	0.238	18.528	0.000	3.942	4.874	-	-	-	-
	Group		0.676	0.668	1.013	0.311	−0.633	1.986	0.676	0.678	−0.483	1.925
	Time	P2	1.426	0.195	7.314	0.000	1.044	1.808	1.426	1.417	1.072	1.793
		P3	1.843	0.197	9.345	0.000	1.456	2.229	1.843	1.834	1.444	2.218
	Subj. stress		−0.262	0.182	−1.441	0.150	−0.618	0.094	−0.262	−0.187	−0.558	0.206
	Group × time × subj. stress	P2	−0.286	0.474	−0.605	0.545	−1.215	0.642	−0.286	−0.379	−1.305	0.439
		P3	−0.555	0.457	−1.213	0.225	−1.451	0.342	−0.555	−0.509	−1.338	0.322
	SPI score		−0.391	0.253	−1.545	0.122	−0.888	0.105	−0.391	−0.390	−0.744	−0.008
*CL*	Intercept		0.660	0.072	9.193	0.000	0.519	0.801	-	-	-	-
	Group		−0.202	0.198	−1.022	0.307	−0.590	0.186	−0.202	−0.209	−0.530	0.091
	Time	P2	−0.124	0.075	−1.659	0.097	−0.271	0.023	−0.124	−0.126	−0.280	0.014
		P3	−0.007	0.076	−0.093	0.926	−0.156	0.142	−0.007	−0.002	−0.143	0.137
	Subj. stress		0.057	0.066	0.873	0.383	−0.071	0.186	0.057	0.052	−0.096	0.189
	Group × time × subj. stress	P2	−0.022	0.179	−0.126	0.900	−0.373	0.328	−0.022	−0.092	−0.558	0.273
		P3	−0.128	0.173	−0.738	0.460	−0.466	0.211	−0.128	−0.154	−0.508	0.222
	SPI score		−0.010	0.069	−0.147	0.883	−0.146	0.125	−0.010	−0.012	−0.109	0.088

No significant effects of group, subjective stress, SPI scores, or their interactions were observed for any of the indices. However, significant effects of time were found: both P2 and P3 predicted increases in dL (P2: β = 1.426, p <.001; P3: β = 1.843, p <.001), indicating improved discriminability and working-memory performance over time. By contrast, no significant effects were observed for the decision criterion (CL).

CL, Decision Criterion (c-like response bias index); dL, Discrimination Index (d′-like sensitivity measure); SPI, Subjective Productivity Interference; Subj. Stress, Subjective Stress level.

### 3.5 Simple regression analysis

**Figure 4:**
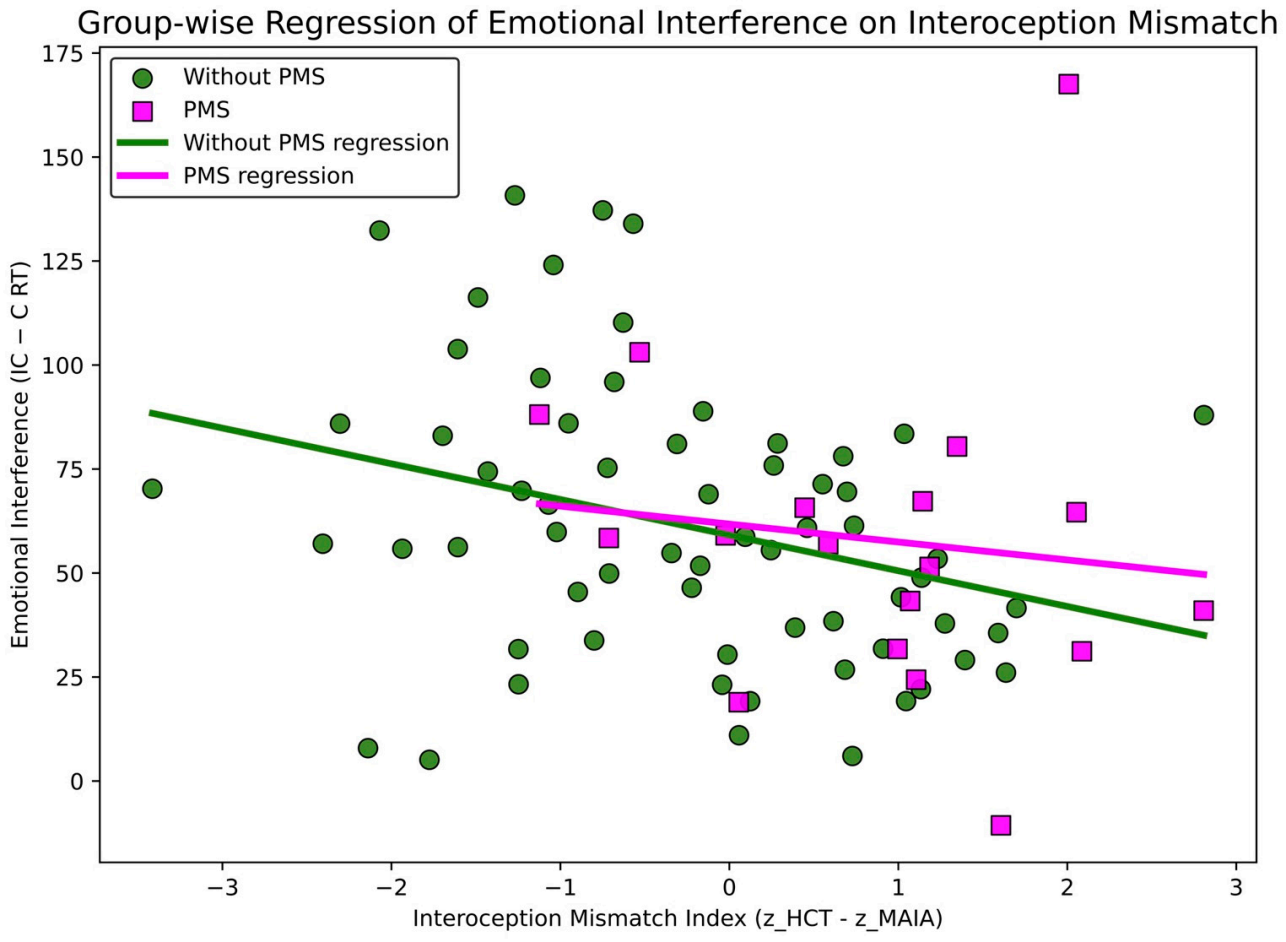
Regression of emotional interference on the interoceptive mismatch index association between the interoceptive mismatch index and emotional interference. A significant negative association was observed overall (*p* =0.016), with no significant group moderation.

A simple linear regression was conducted to predict baseline (P1) emotional interference (Diff_IC–C_RT) from the Interoceptive Mismatch Index (Figure 4). The model was significant [*F*_(1, 81)_ = 6.049, *p* = 0.016], with an *R*^2^ of 0.069.

The Interoceptive Mismatch Index was a significant negative predictor (*b* = −7.162, *SE* = 2.912, *t* = −2.460, *p* = 0.016; standardized β = −0.264), indicating that a larger discrepancy was associated with smaller interference effects (that is, faster reaction times). The Durbin–Watson statistic indicated no autocorrelation in the residuals (*DW* = 2.34); however, normality tests revealed a slight deviation (Omnibus = 10.93, *p* = 0.004; Jarque–Bera = 12.18, *p* = 0.002). This relationship was also significant in Pearson's correlation analysis (*r* = −0.264, *p* = 0.016).

## 4 Discussion

This study aimed to examine how the presence or absence of PMS in the late luteal phase affects women's cognitive functioning. In addition, we investigated how acute stress occurring during the late luteal phase influences cognitive performance. Furthermore, this study conceptualized interoceptive mismatch as a trait-like indicator associated with difficulties in emotion regulation and tested whether this mismatch predicts cognitive control under emotional interference following acute stress, which was operationalized as the reaction-time difference between incongruent and congruent trials (IC–C). Because the experimental design focused on temporal changes across pre-stress, post-stress, and recovery phases, we integrated the analytical results in chronological order, allowing us to evaluate stress effects in a multilayered manner.

Previous research has reported that women with PMS exhibit a mismatch characterized by high interoceptive accuracy and low interoceptive awareness (Suzuki and Ohira, 2025). This finding suggests that bodily signals may influence emotional experience without being sufficiently brought into conscious awareness. Notably, in our previous work (Suzuki and Ohira, 2025), the term *interoceptive awareness* was used; conceptually, this corresponds to *interoceptive sensibility* within the three-dimensional model proposed by Garfinkel et al. (2015).

According to the screening results, 23.6% participants were classified as having PMS (Suzuki and Ohira, 2025). The prevalence of PMS varies substantially depending on sociocultural and demographic factors, making it difficult to establish a consistent estimate (Direkvand-Moghadam et al., 2014). In this context, the ratio of PMS to without-PMS individuals observed in this study (approximately 1:3) may more accurately reflect the actual situation among Japanese university students (Jinko et al., 2021).

PMS was assessed using the PSST, which comprises two major components: the severity of physical and emotional symptoms, and the degree to which PMS interferes with daily functioning (Steiner et al., 2003). Although the PSST does not provide a clinical diagnosis, it is a widely used and validated screening measure (Mirghafourvand et al., 2015; Slyepchenko et al., 2017). Among the PSST items, “work efficiency or productivity” was extracted as the SPI score and included in the analyses. The SPI score was significantly higher in the PMS group, suggesting that subjective performance interference may substantially contribute to diminished daily functioning among women with PMS during the late luteal phase (Supplementary material S1).

### 4.1 Baseline differences in cognition and physiology

Phase 1 was designed to capture baseline (pre-stress) measurements. Regarding cognitive measures, in the Emotional Face–Word Stroop task, no significant differences in reaction time or error rate were found between the PMS and without PMS groups, consistent with previous studies using the same task (Hoyer et al., 2013), but contrasting with studies employing the Emotional Color Stroop task, which reported significant group differences (Eggert et al., 2017).

The Emotional Face–Word Stroop task (Etkin et al., 2006) evaluates higher-order cognitive control by requiring the integration of emotional and semantic information. Therefore, this task is theoretically appropriate for evaluating emotional reactivity and cognitive control instability in individuals with PMS. In contrast, the Emotional Color Stroop task (Eggert et al., 2017) assesses attentional bias toward the visual salience of emotional stimuli. Previous studies using this task have found that women with PMS show increased attentional bias toward negative emotional stimuli.

These two tasks reflect different aspects of cognition. Therefore, PMS-related effects may be more prominent in early-stage attentional processes than in higher-level integrative control. Accordingly, under baseline conditions (that is, in the absence of external stress), PMS-related cognitive vulnerabilities may not be easily detectable and may only emerge in more demanding or emotionally challenging situations.

Furthermore, the luteal phase is associated with increased variability in both cognitive performance and emotional state. Such individual variability may have contributed to the nonsignificant group differences observed in this study phase.

In the 3-Back task, no significant differences in performance—including accuracy, reaction time, and SDT indices—were observed between the PMS and without PMS groups. In contrast, a previous study (Aoki et al., 2022) reported that women with PMS exhibited significantly lower accuracy in both 0-back and 2-back conditions during the luteal phase. This discrepancy may be explained by several methodological differences. For instance, the N-back load differed across studies, and the prior study had smaller group sizes (*N* = 11 per group). Moreover, the participants were from the same university, a population selected through rigorous academic entrance examinations. Given this context, it is possible that baseline differences in cognitive ability had a stronger impact on task performance than PMS-related factors. Indeed, working memory capacity has been positively associated with academic achievement (Almarzouki, 2024).

### 4.2 Stress-induced changes across phases

In the Emotional Face–Word Stroop task, the PMS group exhibited significantly more errors than the without PMS group during the second half of the task (*p* = 0.034). This finding suggests that women with PMS are more prone to making errors when emotional information must be processed under sustained cognitive load. In contrast, no group differences were observed in reaction time. Because both groups showed reduced reaction times across repeated trials, processing speed appears to have been maintained equally in both groups.

The observation that accuracy selectively declined in the PMS group, despite preserved processing speed, indicates a dissociation suggesting a specific cognitive vulnerability in women with PMS. This vulnerability may emerge particularly under conditions of sustained task demands or heightened emotional interference. In other words, such difficulties might not be apparent during rest or under low-load conditions but may become pronounced in the later stages of a task, when stress or emotional load accumulates, potentially compromising sustained attention or interference control.

In contrast, no significant group differences or interactions were found in the 3-Back task. However, significant main effects of Time were observed for hit rate, false alarm rate, correct RT, accuracy, and the SDT sensitivity index *dL* (all *ps* < 0.01 or better), indicating overall improvement in task performance over time. Because this task does not involve emotional interference, it is possible that cognitive resources for working memory and attention were allocated efficiently, thereby attenuating the expression of PMS-related vulnerabilities (Pessoa, 2009). Moreover, the participants in this study were university students with relatively high cognitive abilities, which may also have buffered against performance decline under stress.

### 4.3 Cognitive performance under stress: the role of group and individual predictors

An LMM analysis was conducted to examine the effects of acute stress on cognitive functioning.

In the Emotional Face- Word Stroop task, IC_RTs were positively associated with SPI scores (*p* = 0.040). Participants who reported greater interference with work efficiency or productivity during the premenstrual phase showed longer RTs. This pattern suggests that the correlation between self-assessment (SPI scores) and objective behavioral indicators (IC_RTs), indicating that subjective burden is linked to a decline in actual performance efficiency.

Regarding emotional interference (Diff_IC-C_RT) and attentional bias (Diff_AN-HA_RT), no significant effects of group, time, or psychological markers were observed. These findings suggest that cognitive difficulties under emotional interference are better explained by individual differences in processing speed (captured by SPI), rather than by integration of emotional and semantic information or by biased attention to specific emotional categories.

In the 3-Back task, *dL* showed a significant main effect of Time, with post-stress (P2) and recovery (P3) scores significantly higher than baseline (P1; both *ps* < 0.001). This improvement likely reflects increased task adaptation through repeated trials, enhancing participants' ability to discriminate between targets and non-targets. However, *CL* showed no significant effects of Group, Time, or their interaction, indicating that participants maintained a consistent response strategy (that is, no shift in liberal/conservative bias) across time and groups.

These findings are consistent with the results of the mixed-design ANOVA, which showed significant main effects of Time across multiple indices. These results indicate that overall task performance improved after stress. However, no significant Group or Group × Time interactions were observed, suggesting that the degree of improvement was comparable between the PMS and without PMS groups. Moreover, the consistent *CL* across time and groups indicates a stable decision-making strategy during the 3-Back task, which does not involve emotional interference, reflecting preserved cognitive resilience and efficient resource utilization. Considering that all participants were highly educated university students, it is possible that their high baseline cognitive ability buffered against stress-induced impairments on task performance (Almarzouki, 2024). Overall, tasks with low emotional load, such as the 3-Back task, may be less sensitive to detecting cognitive vulnerabilities associated with PMS.

In our study, both mixed-design ANOVA and LMMs were used to complementarily capture the general patterns of cognitive performance in PMS and the influence of individual physiological differences. The ANOVA results showed that significantly increased error rate was observed only in the Emotional Face-Word Stroop task “hot cognition” among the PMS group, whereas no group differences were found in the 3-Back task “cold cognition.” Furthermore, the LMM demonstrates that individual variability in hot cognition performance is better explained.

Therefore, women with PMS may experience difficulties in processing internal bodily states, including visceral sensations and hormonal changes—that is, interoception—which is essential for emotion generation and self-regulation (Khalsa et al., 2018). Accordingly, we assessed the relationship between Diff_IC–C_RT and the extent of interoceptive mismatch to capture emotion-specific interference.

### 4.4 Interoceptive mismatch and emotional interference

In this study, we defined several information-processing concepts to interpret the association between interoceptive mismatch and emotional interference. Bottom-up processing refers to processes in which attention and emotional responses are automatically elicited based on afferent sensory inputs, including internal bodily signals or facial expressions. Top-down processing refers to higher-order cognitive regulatory mechanisms that modulate or suppress sensory input based on beliefs, expectations, or contextual information. In addition, the term bodily information processing is used to describe the multilayered processes through which bodily inputs and higher-order predictions are integrated to shape emotional and cognitive responses. These conceptual frameworks help clarify the individual differences reflected in interoceptive mismatch.

The Interoceptive Mismatch Index significantly negatively predicted emotional interference (Diff_IC-C_RT) at baseline (P1; *p* = 0.016). Participants with a larger interoceptive discrepancy showed smaller emotional interference. This finding was unexpected, as it contradicts our initial hypothesis that a greater interoceptive discrepancy would impair emotional integration. This inverse relationship can be interpreted by considering that the Interoceptive Mismatch Index and the emotional interference may reflect different cognitive processing styles. Individuals with a positive interoceptive discrepancy may have lower sensibility to their bodily signals and may be inclined to rely more on bottom-up processing, potentially driven by sensory cues such as heartbeats or facial expressions. Conversely, individuals with a negative interoceptive discrepancy may be more inclined to rely on top-down processes, perceiving bodily sensations in ways that may be shaped by beliefs, expectations, or prior knowledge, despite having lower interoceptive accuracy.

The Emotional Face–Word Stroop task requires inhibitory control over the semantic processing of emotional words. Larger emotional interference scores indicate a greater need for top-down cognitive control (Etkin et al., 2011). In contrast, heightened attention to facial expressions may suppress semantic processing of the words and thereby reduce emotional interference, which may reflect the involvement of bottom-up processing driven by sensory inputs such as facial cues. Therefore, this finding that greater interoceptive mismatch was associated with smaller emotional interference may be consistent with the interpretation that interoceptive mismatch is linked to differences in information-processing style (for example, top-down–dominant vs. bottom-up–dominant).

Our previous study suggested that women with PMS tend to exhibit a more positive interoceptive mismatch than those without PMS (Suzuki and Ohira, 2025). This mismatch may be associated with a greater tendency to attend to sensory inputs such as bodily sensations or facial expressions, reflecting a more bottom-up processing style. Such a processing style may make it more difficult to effectively integrate top-down predictions about internal bodily states with actual interoceptive inputs, potentially increasing the discrepancy—or prediction error—between them (Garfinkel et al., 2015). Consequently, women with PMS may be more likely to prioritize sensory inputs (for example, bodily sensations or facial expressions) over semantic information, thereby exhibiting a bottom-up processing tendency. These results suggest that women with PMS tend to show an interoceptive mismatch characterized by greater dominance of bodily signals. This bias may heighten prediction error between the brain's expectations and incoming interoceptive signals. Consequently, the PMS group may be more inclined toward a bottom-up processing style that favors sensory cues over semantic processing.

No significant Group × Interoceptive Mismatch Index interaction was observed, suggesting that this relationship may reflect a general pattern of information processing rather than a PMS-specific characteristic. However, the PMS group demonstrated a more positive interoceptive mismatch than the Without PMS group, indicating that a bottom-up processing style may be more pronounced among women with PMS. These findings suggest that the relationship between emotional interference and interoceptive processing may reflect individual differences in information-processing styles, such as attentional or sensory biases. Elucidating this relationship may deepen our understanding of the neural mechanisms underlying psychopathology and contribute to the development of more personalized assessment and intervention approaches.

Previous studies have reported that interoceptive mismatch—typically conceptualized as a discrepancy between interoceptive accuracy and interoceptive sensibility—is associated with several psychiatric conditions, including anxiety (Garfinkel et al., 2016), depression (Furman et al., 2013), and alexithymia (Garfinkel et al., 2015; Shah et al., 2017). Overall, our findings indicate that PMS may not be solely a mood-related condition but could involve characteristic patterns of bodily information processing. The results further indicate that acute stress may be associated with changes in cognitive–emotional states among women with PMS. In the Emotional Face–Word Stroop task, response speed in inhibitory control of emotional information was comparable between groups, whereas a selective decrease in accuracy was observed in the PMS group. In contrast, stress-related effects were limited in the 3-Back task, which does not involve emotional valence. Although these differences appear subtle, they may accumulate in emotionally demanding daily situations and could contribute to variability in executive functioning.

Interoceptive mismatch was also related to emotional-interference indices, suggesting that interoceptive processing tendencies may become more evident during tasks that require the integration of emotional and cognitive information. Taken together, these patterns imply that the Emotional Face–Word Stroop task may be particularly informative for detecting the behavioral expression of interoceptive processing characteristics under stress.

The findings also suggest that women with PMS may show increased vulnerability in contexts requiring emotion–cognition integration. This vulnerability did not manifest as a broad decline in executive functioning but rather as a selective difficulty in processing emotionally salient conflict. Such a pattern is consistent with theoretical accounts proposing that individuals with heightened emotional sensitivity may be more susceptible to stress-related strain on emotion-regulation systems (Pessoa, 2009).

Finally, the present findings may indicate a tendency toward bottom-up–oriented processing during emotional interference in women with PMS, whereby emotional stimuli are processed relatively quickly compared with semantic stimuli. This pattern was accompanied by a dissociation between interoceptive accuracy and interoceptive sensibility. That is, while women with PMS may perceive bodily reactions and contextual cues, the processes involved in attributing or contextualizing these signals may operate less efficiently, potentially leading to emotional responses that do not fully align with incoming sensory input.

It is important to note that this study included women with PMDD within the PMS group; therefore, some observed characteristics may have been influenced by individuals with PMDD (Henderson et al., 2025). Nevertheless, the finding that interoceptive mismatch predicted emotional interference remains an important observation that may characterize the broader PMS phenotype. PMDD may represent an even more pronounced manifestation of this tendency.

Taken together, these findings suggest that strategies focused on *understanding* and *re-contextualizing* emotional experiences—rather than merely suppressing them—may be more effective for addressing these phenomena.

### 4.5 Implications for clinical practice

From a clinical perspective, interventions that support the reintegration of emotional experiences with bodily responses may be particularly beneficial (Garfinkel et al., 2016; Nord and Garfinkel, 2022). Potential approaches include psychoeducational interventions that promote emotion labeling or reappraisal, mindfulness practices, and emotional reframing techniques that enhance awareness of interoceptive sensations and encourage reinterpretation of bodily responses as meaningful cues about one's internal state.

### 4.6 Implications for education

Regarding educational contexts, there is a growing need for learning and evaluation support that acknowledges physiological rhythms. When educators understand fluctuations in emotional reactivity and attention that commonly occur premenstrually and flexibly adjust teaching approaches or assessment timing, fairness in evaluation may be promoted and psychological burden on students may be reduced.

### 4.7 Implications for the workplace

In workplace settings, institutional support for self-monitoring and environmental adjustment is essential. Rather than pathologizing PMS as a purely mental-health problem, fostering an organizational culture that recognizes PMS as an integrated fluctuation across cognitive, emotional, and bodily domains may be beneficial. Practical strategies include (i) adjusting schedules for critical meetings or cognitively demanding work, (ii) implementing flexible work arrangements aligned with menstrual cycles, and (iii) ensuring psychological safety within teams. Such approaches may enhance individual self-monitoring capacities and promote both productivity and wellbeing.

Across clinical, educational, and workplace applications, a common direction for support is to focus not on *suppressing* bodily responses, but rather on *understanding* and *meaning-making* of those responses. This approach may help alleviate cognitive and emotional instability associated with PMS.

### 4.8 Future directions

PMS should not be viewed merely as an individual concern but as a societal issue that warrants broader attention and action. Understanding its multifaceted nature has the potential to foster more inclusive and supportive environments and to inform the design of comprehensive support strategies.

Future research should focus on the interactions between individual characteristics—such as interoceptive traits and stress sensitivity—and physiological data to deepen our understanding of the mechanisms underlying PMS-related cognitive alterations. Such insights will be essential for all of us to develop more effective interventions and preventive strategies.

### 4.9 Limitations

This study has several limitations. First, PMS was assessed using the PSST, a self-report measure rather than a clinical diagnosis, which limits the diagnostic precision. Therefore, caution is required when generalizing results. Second, the sample sizes between groups were unequal (PMS group: *n* = 21; without PMS group: *n* = 67). Although Welch's *t*-tests were applied to address heterogeneity of variance, ε-corrections were used to adjust for violations of sphericity, and cluster bootstrapping was employed in the mixed-effects models, the ability to detect small effect sizes remains limited. Third, this study focused exclusively on the late luteal phase, preventing comparisons with other menstrual cycle phases. This restricts interpretations regarding cycle specificity and temporal dynamics of PMS-related effects. Finally, the sample consisted solely of female university students, and the reliance on self-report measures may introduce introspective biases. Future research would benefit from incorporating multiple physiological indicators and multi-cycle designs to provide more robust validation.

## 5 Conclusion

This study investigated stress responses and cognitive variability in women with PMS using a multimethod approach. Our results showed that PMS is associated with reduced accuracy in the Emotional Face–Word Stroop task, reflecting heightened susceptibility to emotional interference. By contrast, no group differences were observed in the 3-Back task, indicating that working memory under non-emotional conditions remains preserved. However, both ANOVA and LMM revealed significant time effects on sensitivity (dL), suggesting general performance improvement after stress across all participants. Taken together, these findings suggest that PMS-related cognitive vulnerabilities are more sensitively captured by emotionally charged tasks than by emotionally neutral working memory tasks. Furthermore, the Interoceptive Mismatch Index significantly predicted emotional interference, suggesting a link between bodily signal mismatch and a bottom-up processing style, supporting the hypothesis that PMS-related information processing may favor sensory- and emotion-driven pathways.

Overall, our findings highlight the importance of adopting multidimensional perspectives—interoceptive and self-evaluative—to understand cognitive vulnerability in PMS. Although this study did not provide clear evidence for physiological contributions (Supplementary materials), future research should further investigate this dimension and develop personalized interventions to enhance QoL in affected individuals. Future studies should also include larger and more diverse samples, as PMS prevalence and manifestation vary across individuals and ages. Moreover, assessing multiple menstrual phases would offer a more comprehensive understanding of PMS-related changes. Together, these steps will deepen our longitudinal understanding of PMS and inform better clinical care.

## Data Availability

The original contributions presented in the study are included in the article/Supplementary material, further inquiries can be directed to the corresponding author.
