# Supplementary material for: Women with premenstrual syndrome exhibit bodily information processing and a moderate deficit in emotional interference functioning
Source: Front Psychol. 2026 Jan 12;16:1692811. doi: 10.3389/fpsyg.2025.1692811 (PMC12833253; doi:10.3389/fpsyg.2025.1692811)
Supplement: Supplementary file 1 [file Data_Sheet_1.zip › Supplementary Material/Supplementary Material S1.DOCX]

Supplementary Material

***Supplementary Material S1.***

***Supplementary Figure S1. Distribution of Subjective Performance Interference (SPI) Scores by Group***

Violin plots illustrating the distribution of Subjective Performance Interference (SPI) scores between women with PMS and without PMS.

The width of each violin represents the density distribution of SPI scores within each group.

Central black bars indicate the median and interquartile range.

Women with PMS showed higher SPI scores compared to those without PMS, indicating greater self-reported interference with work efficiency or productivity during the premenstrual phase.
