# Supplementary material for: Women with premenstrual syndrome exhibit bodily information processing and a moderate deficit in emotional interference functioning
Source: Front Psychol. 2026 Jan 12;16:1692811. doi: 10.3389/fpsyg.2025.1692811 (PMC12833253; doi:10.3389/fpsyg.2025.1692811)

Supplementary Material

***Supplementary Material S2***

***Salivary cortisol measurements***

Saliva samples were collected using the unstimulated passive drool method. Participants were instructed to rinse their mouths with water 10 min before collection and to refrain from eating or drinking thereafter. Samples were refrigerated at 4℃ within 10 min of collection and stored at -20℃ or below until assay. Participants were also instructed to avoid saliva collection within 60 min of eating or within 12 h of alcohol consumption.

After complete thawing, samples were vortexed and centrifuged at 1,500 × *g* for 15 min to remove mucus and particulate matter. The supernatant was equilibrated to 22–25℃ and aliquoted into assay plates. To maintain sample integrity, repeated freeze-thaw cycles were avoided. If necessary, additional centrifugation was performed.

For cortisol assay, salivary cortisol concentrations (Cort conc) were measured using the Salimetrics High Sensitivity Salivary Cortisol ELISA Kit (Item No. 1-3002, Single 96-Well Kit). According to the manufacturer's specifications, the assay has a lower limit of sensitivity of 0.007 μg/dL, with an intra-assay coefficient of variation (CV) averaging 4.6% and an inter-assay CV averaging 6.0%. The kit utilizes a monoclonal antibody specific to cortisol, with negligible cross-reactivity with other steroid hormones (<0.1%).

Samples with a coefficient of variation >15% were excluded from the analysis.

Saliva samples were collected at four time points:

- P1 (Baseline): ~10:30-11:00 a.m.
- P2-immediate (immediately post-stress): ~11:30-12:00 p.m.
- P2-late (25-30 min post-stress): ~12:00-12:30 p.m.
- P3 (Recovery): ~12:30-1:00 p.m.

Although saliva collection was originally planned during the WT phase (30–60 min post-stress), it was conducted at the end of P2 (25–30 min post-stress) to capture the delayed cortisol peak. Therefore, P2-late was therefore treated as an additional time point in the ANOVA. During the WT phase, participants completed only questionnaires.

***Data analysis for cortisol concentration***

To assess effects of temporal changes in stress, mixed-design analyses of variance (ANOVAs) were performed. For Cort conc, Group (PMS vs. Without PMS) was treated as a between-subject factor, and Time (pre-stress baseline [P1], immediately after stress [P2-immediate], post-waiting period [P2-late], and recovery [P3]) was treated as a within-subject factor.

Cortisol change scores (Δcortisol) were calculated as ΔCortisol-immediate response (P2-immediate - P1), ΔCortisol-peak response (P2-late - P1), and ΔCortisol-recovery response (P3 - P1), and were included in the analysis.

***Results***

Mixed-design ANOVAs on Cort conc and ΔCortisol revealed no significant main effect of Group (PMS vs. Without PMS) and no Group × Time interactions (all *ps* > .05). However, a significant main effect of Time was observed, indicating a gradual decrease in Cort conc over time, *F*(2.28, 182.4) = 7.655, *η*² = .087, *p* < .001. Similarly, for ΔCortisol, a significant main effect of Time was also found, *F*(1.74, 139.4) = 10.089, *η*² = .112, *p* < .001.

An exploratory post-hoc analysis revealed a significant decline in Cort conc from P1 to P3 (*d* = .738, adjusted *p* = .028), and from P2_immediate to P3 (*d* = .564, adjusted *p* = .048). Although the Without PMS group showed a similar trend, none of the pairwise comparisons reached statistical significance after Holm correction.

**Supplementary Figure 1a. Group differences in salivary cortisol concentration**  The triangular arrow indicates stress induction.

stress induction

**Supplementary Figure 1b.** **Group differences in ΔCortisol responses;** ΔCortisol-immediate (P2-immediate − P1), ΔCortisol-peak (P2-late − P1), and ΔCortisol-recovery (P3 − P1) responses


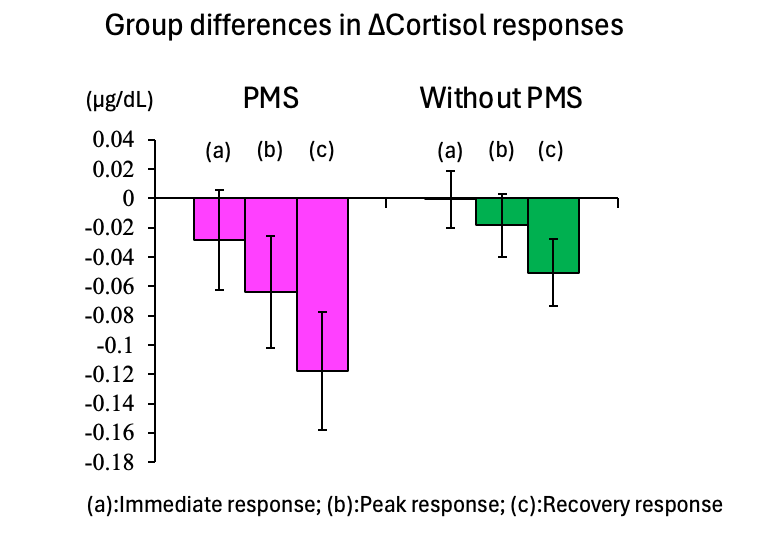

Supplement: Supplementary file 1 [file Data_Sheet_1.zip › Supplementary Material/Supplementary Material S2.DOCX]
