# Supplementary material for: Women with premenstrual syndrome exhibit bodily information processing and a moderate deficit in emotional interference functioning
Source: Front Psychol. 2026 Jan 12;16:1692811. doi: 10.3389/fpsyg.2025.1692811 (PMC12833253; doi:10.3389/fpsyg.2025.1692811)
Supplement: Supplementary file 1 [file Data_Sheet_1.zip › Supplementary Material/Supplementary Material S3.DOCX]

Supplementary Material

***Supplementary Material S3. Results of t-tests for the Emotional Face-Word Stroop and 3-Back tasks.***

**Supplementary Table. Results of t-tests for the Emotional Face–Word Stroop and 3-Back tasks.**

Independent-samples t-tests were conducted to compare the PMS and without-PMS groups for each index at baseline (P1). Effect sizes are reported as Cohen’s *d* with 95% confidence intervals. Significance codes: *p* < .05 (*), *p* < .01 (), *p* < .001 (*), *n.s.* = not significant.

**Abbreviations Used in Cognitive Tasks**

**Emotional Face–Word Stroop task abbreviations:**

C = Congruent (facial expression and emotional word are matched); IC = Incongruent (facial expression and emotional word are mismatched); HA = Happy (positive facial expression); AN = Angry (negative facial expression); RT = Reaction Time; Error = Error rate; FH / SH = the first and second halves of the same trial sequence within the task**.**

**3-Back task abbreviations:**

Accuracy rate = proportion of correct responses; HIT rate = proportion of Hits among signal trials; FalseAlarm rate = proportion of False Alarms among noise trials; Empty rate = proportion of trials with no response; Correct RT = mean reaction time for correct responses (Hits and Correct Rejections);  FH / SH = the first and second halves of the same trial sequence within the task; *dL* = sensitivity index based on signal detection theory; *CL* = decision criterion based on signal detection theory.

**The SPI score** represents the Subjective Performance Interference score.

| Indices | Group | Mean | *SD* | *SE* | 95% *CI*  (Lower) | 95% *CI*  (Upper) | *t (df)* | *p*-value | Cohen's *d* | Significant |
| --- | --- | --- | --- | --- | --- | --- | --- | --- | --- | --- |
| ***Emotional Face-Word Stroop task*** | | | | | | | | | | |
| C_RT | PMS | 584.737 | 77.89 | 17.869 | 549.196 | 620.279 | *t*(27.031) = 0.482 | 0.633 | 0.132 | *n.s.* |
|  | Without PMS | 575.158 | 70.34 | 8.658 | 557.937 | 592.379 |  |  |  |  |
| C_C_RT | PMS | 580.75 | 76.479 | 17.545 | 545.853 | 615.648 | *t*(27.573) = 0.410 | 0.685 | 0.11 | *n.s.* |
|  | Without PMS | 572.714 | 71.01 | 8.741 | 555.329 | 590.099 |  |  |  |  |
| IC_C_RT | PMS | 589.44 | 85.405 | 19.593 | 550.47 | 628.41 | *t*(26.176) = 0.522 | 0.606 | 0.146 | *n.s.* |
|  | Without PMS | 578.176 | 73.549 | 9.053 | 560.17 | 596.183 |  |  |  |  |
| IC_RT | PMS | 642.579 | 95.645 | 21.943 | 598.936 | 686.222 | *t*(25.787) = 0.243 | 0.81 | 0.069 | *n.s.* |
|  | Without PMS | 636.738 | 80.471 | 9.905 | 617.037 | 656.439 |  |  |  |  |
| C_IC_RT | PMS | 638.434 | 94.319 | 21.638 | 595.396 | 681.471 | *t*(25.830) = 0.283 | 0.779 | 0.08 | *n.s.* |
|  | Without PMS | 631.712 | 79.563 | 9.794 | 612.233 | 651.191 |  |  |  |  |
| IC_IC_RT | PMS | 650.545 | 104.468 | 23.967 | 602.876 | 698.213 | *t*(25.971) = 0.150 | 0.882 | 0.042 | *n.s.* |
|  | Without PMS | 646.596 | 88.878 | 10.94 | 624.836 | 668.355 |  |  |  |  |
| C_Error | PMS | 1.105 | 1.197 | 0.275 | 0.559 | 1.651 | *t*(25.134) = 0.605 | 0.551 | 0.176 | *n.s.* |
|  | Without PMS | 0.924 | 0.966 | 0.119 | 0.688 | 1.161 |  |  |  |  |
| C_C_Error | PMS | 0.842 | 0.958 | 0.22 | 0.405 | 1.279 | *t*(27.149) = 0.841 | 0.408 | 0.229 | *n.s.* |
|  | Without PMS | 0.636 | 0.871 | 0.107 | 0.423 | 0.85 |  |  |  |  |
| IC_C_Error | PMS | 0.263 | 0.452 | 0.104 | 0.057 | 0.47 | *t*(34.712) = −0.200 | 0.843 | -0.046 | *n.s.* |
|  | Without PMS | 0.288 | 0.548 | 0.067 | 0.154 | 0.422 |  |  |  |  |
| IC_Error | PMS | 2.158 | 2.115 | 0.485 | 1.193 | 3.123 | *t*(24.318) = 0.590 | 0.56 | 0.177 | *n.s.* |
|  | Without PMS | 1.848 | 1.61 | 0.198 | 1.454 | 2.243 |  |  |  |  |
| C_IC_Error | PMS | 1.737 | 1.593 | 0.365 | 1.01 | 2.464 | *t*(25.223) = 1.012 | 0.321 | 0.293 | *n.s.* |
|  | Without PMS | 1.333 | 1.293 | 0.159 | 1.017 | 1.65 |  |  |  |  |
| IC_IC_Error | PMS | 0.421 | 0.692 | 0.159 | 0.105 | 0.737 | *t*(28.928) = −0.523 | 0.605 | -0.136 | *n.s.* |
|  | Without PMS | 0.515 | 0.685 | 0.084 | 0.347 | 0.683 |  |  |  |  |
| HA_RT | PMS | 601.923 | 85.167 | 19.539 | 563.062 | 640.785 | *t*(26.355) = 0.468 | 0.644 | 0.13 | *n.s.* |
|  | Without PMS | 591.831 | 74.107 | 9.122 | 573.688 | 609.974 |  |  |  |  |
| HA_C_RT | PMS | 583.341 | 80.477 | 18.463 | 546.619 | 620.062 | *t*(26.948) = 0.593 | 0.558 | 0.162 | *n.s.* |
|  | Without PMS | 571.178 | 72.356 | 8.906 | 553.464 | 588.892 |  |  |  |  |
| HA_IC_RT | PMS | 656.189 | 105.622 | 24.231 | 607.994 | 704.384 | *t*(25.897) = 0.147 | 0.885 | 0.041 | *n.s.* |
|  | Without PMS | 652.288 | 89.46 | 11.012 | 630.386 | 674.19 |  |  |  |  |
| AN_RT | PMS | 601.029 | 81.171 | 18.622 | 563.991 | 638.067 | *t*(27.304) = 0.321 | 0.751 | 0.087 | *n.s.* |
|  | Without PMS | 594.374 | 74.348 | 9.152 | 576.172 | 612.576 |  |  |  |  |
| AN_C_RT | PMS | 586.259 | 77.268 | 17.727 | 551.001 | 621.516 | *t*(28.338) = 0.329 | 0.744 | 0.087 | *n.s.* |
|  | Without PMS | 579.684 | 74.42 | 9.16 | 561.465 | 597.904 |  |  |  |  |
| AN_IC_RT | PMS | 632.074 | 93.635 | 21.481 | 589.349 | 674.8 | *t*(26.005) = 0.301 | 0.765 | 0.085 | *n.s.* |
|  | Without PMS | 624.954 | 79.827 | 9.826 | 605.41 | 644.497 |  |  |  |  |
| HA_Error | PMS | 1.842 | 1.642 | 0.377 | 1.093 | 2.591 | *t*(24.510) = 0.876 | 0.389 | 0.261 | *n.s.* |
|  | Without PMS | 1.485 | 1.268 | 0.156 | 1.174 | 1.795 |  |  |  |  |
| HA_C_Error | PMS | 0.632 | 0.761 | 0.175 | 0.284 | 0.979 | *t*(25.061) = 1.011 | 0.322 | 0.295 | *n.s.* |
|  | Without PMS | 0.439 | 0.611 | 0.075 | 0.29 | 0.589 |  |  |  |  |
| HA_IC_Error | PMS | 1.211 | 1.273 | 0.292 | 0.63 | 1.791 | *t*(26.481) = 0.512 | 0.613 | 0.142 | *n.s.* |
|  | Without PMS | 1.045 | 1.115 | 0.137 | 0.772 | 1.319 |  |  |  |  |
| AN_Error | PMS | 1.421 | 1.305 | 0.299 | 0.826 | 2.016 | *t*(28.859) = 0.393 | 0.697 | 0.102 | *n.s.* |
|  | Without PMS | 1.288 | 1.286 | 0.158 | 0.973 | 1.603 |  |  |  |  |
| AN_C_Error | PMS | 0.474 | 0.697 | 0.16 | 0.156 | 0.792 | *t*(29.534) = −0.061 | 0.951 | -0.016 | *n.s.* |
|  | Without PMS | 0.485 | 0.707 | 0.087 | 0.312 | 0.658 |  |  |  |  |
| AN_IC_Error | PMS | 0.947 | 1.079 | 0.247 | 0.455 | 1.44 | *t*(27.762) = 0.521 | 0.606 | 0.139 | *n.s.* |
|  | Without PMS | 0.803 | 1.011 | 0.124 | 0.556 | 1.051 |  |  |  |  |
| C_RT_FH | PMS | 583.414 | 80.872 | 18.553 | 546.512 | 620.316 | *t*(26.165) = 0.669 | 0.509 | 0.188 | *n.s.* |
|  | Without PMS | 569.734 | 69.601 | 8.567 | 552.694 | 586.774 |  |  |  |  |
| IC_RT_FH | PMS | 657.288 | 104.348 | 23.939 | 609.674 | 704.902 | *t*(23.996) = 1.069 | 0.296 | 0.325 | *n.s.* |
|  | Without PMS | 629.742 | 77.454 | 9.534 | 610.78 | 648.705 |  |  |  |  |
| Error_FH | PMS | 1.421 | 1.305 | 0.299 | 0.826 | 2.016 | *t*(28.408) = 0.170 | 0.866 | 0.045 | *n.s.* |
|  | Without PMS | 1.364 | 1.26 | 0.155 | 1.055 | 1.672 |  |  |  |  |
| C_RT_SH | PMS | 613.883 | 86.181 | 19.771 | 574.559 | 653.208 | *t*(27.787) = 0.297 | 0.769 | 0.079 | *n.s.* |
|  | Without PMS | 607.309 | 80.867 | 9.954 | 587.51 | 627.107 |  |  |  |  |
| IC_RT_SH | PMS | 626.358 | 98.463 | 22.589 | 581.43 | 671.286 | *t*(28.771) = -0.686 | 0.498 | -0.179 | *n.s.* |
|  | Without PMS | 643.883 | 96.707 | 11.904 | 620.207 | 667.559 |  |  |  |  |
| Error_SH | PMS | 1.842 | 1.463 | 0.336 | 1.175 | 2.51 | *t*(27.909) = 1.151 | 0.26 | 0.307 | *n.s.* |
|  | Without PMS | 1.409 | 1.381 | 0.17 | 1.071 | 1.747 |  |  |  |  |
| Diff_EST_  IC-C_RT | PMS | 59.64 | 37.124 | 8.301 | 43.132 | 76.148 | *t*(29.357) = -0.209 | 0.836 | -0.055 | *n.s.* |
|  | Without PMS | 61.581 | 34.039 | 4.19 | 53.248 | 69.913 |  |  |  |  |
| Diff_EST_  AN-HA_RT | PMS | -0.894 | 30.248 | 6.939 | -14.697 | 12.908 | *t*(35.112) = -0.414 | 0.681 | -0.095 | *n.s.* |
|  | Without PMS | 2.543 | 37.069 | 4.563 | -6.532 | 11.619 |  |  |  |  |
| ***3-Back task*** | | | | | | | | | | |
| Accuracy rate | PMS | 0.893 | 0.066 | 0.015 | 0.864 | 0.922 | *t*(30.709) = 0.148 | 0.884 | 0.038 | *n.s.* |
|  | Without PMS | 0.891 | 0.064 | 0.008 | 0.875 | 0.906 |  |  |  |  |
| HIT rate | PMS | 0.831 | 0.095 | 0.021 | 0.788 | 0.873 | *t*(38.867) =1.065 | 0.294 | 0.239 | *n.s.* |
|  | Without PMS | 0.803 | 0.119 | 0.015 | 0.774 | 0.832 |  |  |  |  |
| FalseAlarm rate | PMS | 0.074 | 0.069 | 0.015 | 0.043 | 0.105 | *t*(26.223) = 0.513 | 0.612 | 0.149 | *n.s.* |
|  | Without PMS | 0.065 | 0.053 | 0.007 | 0.052 | 0.078 |  |  |  |  |
| Empty rate | PMS | 0.153 | 0.123 | 0.028 | 0.098 | 0.207 | *t*(37.690) =0.895 | 0.377 | 0.204 | *n.s.* |
|  | Without PMS | 0.123 | 0.15 | 0.018 | 0.086 | 0.16 |  |  |  |  |
| Correct RT | PMS | 865.874 | 284.48 | 63.612 | 739.375 | 992.372 | *t*(28.404) = 0.010 | 0..992 | 0.003 | *n.s.* |
|  | Without PMS | 865.161 | 249.074 | 30.659 | 804.192 | 926.129 |  |  |  |  |
| Accuracy rate_FH | PMS | 0.787 | 0.111 | 0.025 | 0.736 | 0.837 | *t*(37.897) = 0.484 | 0.631 | 0.107 | *n.s.* |
|  | Without PMS | 0.772 | 0.146 | 0.018 | 0.736 | 0.808 |  |  |  |  |
| Correct RT_FH | PMS | 0.919 | 0.329 | 0.075 | 0.769 | 1.069 | *t*(25.778) = 0.052 | 0.959 | 0.015 | *n.s.* |
|  | Without PMS | 0.915 | 0.277 | 0.034 | 0.847 | 0.982 |  |  |  |  |
| Accuracy rate_SH | PMS | 0.714 | 0.204 | 0.047 | 0.621 | 0.807 | *t*(24.000) = -1.331 | 0.196 | -0.404 | *n.s.* |
|  | Without PMS | 0.781 | 0.151 | 0.019 | 0.744 | 0.818 |  |  |  |  |
| Correct RT_SH | PMS | 0.796 | 0.27 | 0.062 | 0.673 | 0.919 | *t*(27.391) = -0.311 | 0.758 | -0.084 | *n.s.* |
|  | Without PMS | 0.817 | 0.248 | 0.031 | 0.756 | 0.878 |  |  |  |  |
| *dL* | PMS | 4.688 | 1.675 | 0.374 | 3.944 | 5.433 | *t*(32.005) = 0.157 | 0.876 | 0.039 | *n.s.* |
|  | Without PMS | 4.621 | 1.712 | 0.211 | 4.202 | 5.04 |  |  |  |  |
| *CL* | PMS | 0.516 | 0.489 | 0.109 | 0.299 | 0.734 | *t*(29.271) = -1.110 | 0.276 | -0.295 | *n.s.* |
|  | Without PMS | 0.652 | 0.446 | 0.055 | 0.543 | 0.761 |  |  |  |  |
| ***Subjective Performance Interference*** | | | | | | | | | | |
| SPI score | PMS | 3.1 | 0.641 | 0.143 | 2.815 | 3.385 | *t*(27.180) = 10.275 | .000 | 2.868 | *** |
|  | Without PMS | 1.485 | 0.532 | 0.065 | 1.357 | 1.614 |  |  |  |  |
