# Supplementary material for: Women with premenstrual syndrome exhibit bodily information processing and a moderate deficit in emotional interference functioning
Source: Front Psychol. 2026 Jan 12;16:1692811. doi: 10.3389/fpsyg.2025.1692811 (PMC12833253; doi:10.3389/fpsyg.2025.1692811)
Supplement: Supplementary file 1 [file Data_Sheet_1.zip › Supplementary Material/Supplementary Material S4.DOCX]

Supplementary Material

***Supplementary Material S4. Results of mixed-design ANOVAs for the Emotional Face-Word Stroop and 3-Back tasks.***

Mixed-design ANOVAs were conducted for each index with Group (PMS vs. without PMS) as a between-subject factor and Time (P1, P2, P3) as a within-subject factor. Effect sizes are partial *η*² with 95% confidence intervals. Significance codes: *p* < .05 (*), *p* < .01 (**), *p* < .001 (***), *n.s.* = not significant.

**Abbreviations Used in Cognitive Tasks**

**Emotional Face–Word Stroop task abbreviations:**

C = *Congruent* (facial expression and emotional word match);

IC = *Incongruent* (facial expression and emotional word mismatch);

HA = *Happy* (positive facial expression);

AN = *Angry* (negative facial expression);

RT = *Reaction Time*;

Error = *Error rate*;

FirstHalf / SecondHalf = the first and second halves of the trial sequence within the task.

**3-Back task abbreviations:**

Accuracy rate = proportion of correct responses;

HIT rate = proportion of Hits among signal trials;

FalseAlarm rate = proportion of False Alarms among noise trials;

Empty rate = proportion of trials with no response;

Correct RT = mean reaction time for correct responses (Hits and Correct Rejections);

dL = *sensitivity* index (signal detection theory);

CL = *decision criterion* (signal detection theory).

| Indices | Effect | Effect size  (*η2*) | 95%*CI* | *F (df)* | *p*-value | Significant |
| --- | --- | --- | --- | --- | --- | --- |
| **Emotional Stroop Task** | | | | | | |
| C_RT | Group | .002 | .000, .060 | *F*(1,83)=0.171 | .680 | *n.s*. |
|  | Time | .072 | --- | *F*(2,166)=6.433 | .002 | ** |
|  | Interaction | .002 | --- | *F(*2,166)=0.164 | .839 | *n.s.* |
| IC_RT | Group | .004 | .000, .070 | *F*(1,83)=0.351 | .555 | *n.s.* |
|  | Time | .059 | --- | *F(*2,166)=5.223 | .007 | ** |
|  | Interaction | .009 | --- | *F*(2,166)=0.713 | .490 | *n.s.* |
| C-C_RT | Group | .001 | .000, .054 | *F*(1,83)=0.109 | .742 | *n.s.* |
|  | Time | .063 | --- | *F*(2,166)=5.623 | .005 | ** |
|  | Interaction | .002 | --- | *F*(2,166)=0.156 | .850 | *n.s.* |
| IC-C_RT | Group | .004 | .000, .071 | *F*(1,83)=0.373 | .543 | *n.s.* |
|  | Time | .064 | --- | *F*(2,166)=5.721 | .004 | ** |
|  | Interaction | .000 | --- | *F*(2,166)=0.039 | .958 | *n.s.* |
| C-IC_RT | Group | .005 | .000, .072 | *F*(1,83)=0.387 | .535 | *n.s.* |
|  | Time | .028 | --- | *F*(2,166)=2.403 | .094 | *n.s.* |
|  | Interaction | .009 | --- | *F*(2,166)=0.736 | .481 | *n.s.* |
| IC-IC_RT | Group | .004 | .000, .067 | *F*(1,83)=0.292 | .591 | *n.s.* |
|  | Time | .119 | --- | *F*(2,166)=11.235 | .000 | *** |
|  | Interaction | .005 | --- | *F*(2,166)=0.437 | .643 | *n.s.* |
| C_Error | Group | .028 | .000, .127 | *F*(1,84)=2.454 | .121 | *n.s.* |
|  | Time | .057 | --- | *F*(2,168)=5.038 | .026 | * |
|  | Interaction | .026 | --- | *F*(2,168)=2.240 | .137 | *n.s.* |
| C-C_Error | Group | .029 | .000, .129 | *F*(1,84)=2.541 | .115 | *n.s.* |
|  | Time | .071 | --- | *F*(2,168)=6.371 | .002 | ** |
|  | Interaction | .011 | --- | *F*(2,168)=0.960 | .385 | *n.s.* |
| IC-C_Error | Group | .003 | .000, .063 | *F*(1,84)=0.224 | .637 | *n.s.* |
|  | Time | .000 | --- | *F*(2,168)=0.002 | .967 | *n.s.* |
|  | Interaction | .009 | --- | *F*(2,168)=0.743 | .391 | *n.s.* |
| IC_Error | Group | .018 | .000, .106 | *F*(1,84)=1.498 | .224 | *n.s.* |
|  | Time | .115 | --- | *F*(2,168)=10.955 | .001 | ** |
|  | Interaction | .012 | --- | *F*(2,168)=1.055 | .307 | *n.s.* |
| C-IC_Error | Group | .021 | .000, .113 | *F*(1,84)=1.826 | .180 | *n.s.* |
|  | Time | .047 | --- | *F*(2,168)=4.138 | .045 | * |
|  | Interaction | .003 | --- | *F*(2,168)=0.249 | .619 | *n.s.* |
| IC-IC_Error | Group | .002 | .000, .059 | *F*(1,84)=0.172 | .679 | *n.s.* |
|  | Time | .085 | --- | *F*(2,168)=7.777 | .007 | ** |
|  | Interaction | .014 | --- | *F*(2,168)=1.190 | .279 | *n.s.* |
| HA_RT | Group | .002 | .000, .061 | *F*(1,83)=0.187 | .667 | *n.s.* |
|  | Time | .100 | --- | *F*(2,166)=9.205 | .000 | *** |
|  | Interaction | .001 | --- | *F*(2,166)=0.055 | .939 | *n.s.* |
| HA_C_RT | Group | .004 | .000, .068 | *F*(1,83)=0.298 | .586 | *n.s.* |
|  | Time | .087 | --- | *F*(2,166)=7.950 | .001 | ** |
|  | Interaction | .001 | --- | *F*(2,166)=0.048 | .946 | *n.s.* |
| HA_IC_RT | Group | .001 | .000, .045 | *F*(1,83)=0.052 | .820 | *n.s.* |
|  | Time | .075 | --- | *F*(2,166)=6.736 | .002 | **** |
|  | Interaction | .000 | --- | *F*(2,166)=0.041 | .958 | *n.s.* |
| AN_RT | Group | .003 | .000, .063 | *F*(1,83)=0.212 | .646 | *n.s.* |
|  | Time | .053 | --- | *F*(2,166)=4.667 | .011 | * |
|  | Interaction | .007 | --- | *F*(2,166)=0.613 | .539 | *n.s.* |
| AN_C_RT | Group | .001 | .000, .047 | *F*(1,83)=0.062 | .804 | *n.s.* |
|  | Time | .055 | --- | *F*(2,166)=4.798 | .010 | ** |
|  | Interaction | .004 | --- | *F*(2,166)=0.369 | .690 | *n.s.* |
| AN_IC_RT | Group | .009 | .000, .086 | *F*(1,83)=0.748 | .390 | *n.s.* |
|  | Time | .032 | --- | *F*(2,166)=2.760 | .066 | *n.s.* |
|  | Interaction | .017 | --- | *F*(2,166)=1.468 | .233 | *n.s.* |
| HA_Error | Group | .038 | .000, .144 | *F*(1,84)=3.338 | .071 | *n.s.* |
|  | Time | .069 | --- | *F*(2,168)=6.212 | .015 | * |
|  | Interaction | .019 | --- | *F*(2,168)=1.642 | .204 | *n.s.* |
| HA_C_Error | Group | .010 | .000, .087 | *F*(1,84)=0.818 | .368 | *n.s.* |
|  | Time | .049 | --- | *F*(2,168)=4.295 | .041 | * |
|  | Interaction | .001 | --- | *F*(2,168)=0.048 | .826 | *n.s.* |
| HA_IC_Error | Group | .037 | .000, .142 | *F*(1,84)=3.242 | .075 | *n.s.* |
|  | Time | .030 | --- | *F*(2,168)=2.602 | .111 | *n.s.* |
|  | Interaction | .024 | --- | *F*(2,168)=2.087 | .152 | *n.s.* |
| AN_Error | Group | .010 | .000, .089 | *F*(1,84)=0.885 | .350 | *n.s.* |
|  | Time | .082 | --- | *F*(2,168)=7.492 | .008 | ** |
|  | Interaction | .010 | --- | *F*(2,168)=0.810 | .371 | *n.s.* |
| AN_C_Error | Group | .033 | .000, .135 | *F*(1,84)=2.862 | .094 | *n.s.* |
|  | Time | .014 | --- | *F*(2,168)=1.170 | .283 | *n.s.* |
|  | Interaction | .039 | --- | *F*(2,168)=3.387 | .069 | *n.s.* |
| AN_IC_Error | Group | .001 | .000, .044 | *F*(1,84)=0.049 | .826 | *n.s.* |
|  | Time | .073 | --- | *F*(2,168)=6.623 | .002 | ** |
|  | Interaction | .001 | --- | *F*(2,168)=0.043 | .958 | *n.s.* |
| C_RT_FH | Group | .004 | .000, .070 | *F*(1,83)=0.342 | .560 | *n.s.* |
|  | Time | .086 | --- | *F*(2,166)=7.795 | .001 | ** |
|  | Interaction | .002 | --- | *F*(2,166)=0.199 | .820 | *n.s.* |
| IC_RT_FH | Group | .011 | .000, .091 | *F*(1,83)=0.909 | .343 | *n.s.* |
|  | Time | .119 | --- | *F*(2,166)=11.202 | .000 | *** |
|  | Interaction | .003 | --- | *F*(2,166)=0.256 | .771 | *n.s.* |
| Error_FH | Group | .039 | .000, .147 | *F*(1,83)=3.408 | .068 | *n.s.* |
|  | Time | .075 | --- | *F*(2,166)=6.724 | .002 | ** |
|  | Interaction | .023 | --- | *F*(2,166)=1.923 | .150 | *n.s.* |
| C_RT_SH | Group | .001 | .000, .052 | *F*(1,83)=0.093 | .761 | *n.s.* |
|  | Time | .165 | --- | *F*(2,166)=16.417 | .000 | *** |
|  | Interaction | .004 | --- | *F*(2,166)=0.299 | .730 | *n.s.* |
| IC_RT_SH | Group | .000 | .000, .015 | *F*(1,83)=0.004 | .952 | *n.s.* |
|  | Time | .000 | --- | *F*(2,166)=0.031 | .915 | *n.s.* |
|  | Interaction | .005 | --- | *F*(2,166)=0.381 | .596 | *n.s.* |
| Error_SH | Group | .060 | .000, .178 | *F*(1,83)=5.335 | .023 | * |
|  | Time | .024 | --- | *F*(2,166)=2.024 | .135 | *n.s.* |
|  | Interaction | .011 | --- | *F*(2,166)=0.925 | .399 | *n.s.* |
| Diff_IC-C_RT | Group | .005 | .000, .074 | *F*(1,83)=0.440 | .509 | *n.s.* |
|  | Time | .001 | --- | *F*(2,166)=0.105 | .901 | *n.s.* |
|  | Interaction | .018 | --- | *F*(2,166)=1.538 | .218 | *n.s.* |
| Diff_AN-HA_RT | Group | .000 | .000, .027 | *F*(1,83)=0.010 | .920 | *n.s.* |
|  | Time | .092 | --- | *F*(2,166)=8.448 | .000 | ** |
|  | Interaction | .014 | --- | *F*(2,166)=1.191 | .306 | *n.s.* |
| **3-Back Task** | | | | | | |
| Accuracy rate | Group | .001 | .000, .050 | *F*(1,83) = 0.082 | .776 | *n.s.* |
|  | Time | .286 | --- | *F*(2,166) = 33.229 | .000 | *** |
|  | Interaction | .005 | --- | *F*(2,166) = 0.439 | .620 | *n.s.* |
| HIT rate | Group | .002 | .000, .060 | *F*(1,83) = 0.179 | .673 | *n.s.* |
|  | Time | .170 | --- | *F*(2,166) = 16.972 | .000 | *** |
|  | Interaction | .009 | --- | *F*(2,166) = 0.748 | .471 | *n.s.* |
| False Alarm rate | Group | .013 | .000, .097 | *F*(1,83) = 1.132 | .290 | *n.s.* |
|  | Time | .213 | --- | *F*(1,166) = 22.423 | .000 | *** |
|  | Interaction | .000 | --- | *F*(2,166) = 0.015 | .970 | *n.s.* |
| Empty rate | Group | .004 | .000, .071 | *F*(1,83) = 0.362 | .549 | *n.s.* |
|  | Time | .023 | --- | *F*(2,166) = 1.962 | .157 | *n.s.* |
|  | Interaction | .004 | --- | *F*(2,166) = 0.327 | .649 | *n.s.* |
| Correct_RT | Group | .000 | .000, .032 | *F*(1,83) = 0.017 | .897 | *n.s.* |
|  | Time | .324 | --- | *F*(2,166) = 39.849 | .000 | *** |
|  | Interaction | .003 | --- | *F*(2,166) = 0.239 | .715 | *n.s.* |
| Accuracy rate_FH | Group | .002 | .000, .058 | *F*(1,81) = 0.142 | .708 | *n.s.* |
|  | Time | .003 | --- | *F*(162) = 0.230 | .758 | *n.s.* |
|  | Interaction | .011 | --- | *F*(162) = 0.901 | .394 | *n.s.* |
| Correct_RT_FH | Group | .000 | .000, .027 | *F*(1,80) = 0.010 | .922 | *n.s.* |
|  | Time | .323 | --- | *F*(2,160) = 38.170 | .000 | **** |
|  | Interaction | .002 | --- | *F*(2,160) = 0.122 | .835 | *n.s.* |
| Accuracy rate_SH | Group | .007 | .000, .082 | *F*(1,81) = 0.598 | .441 | *n.s.* |
|  | Time | .030 | --- | *F*(2,162) = 2.526 | .097 | *n.s.* |
|  | Interaction | .013 | --- | *F*(2,162) = 1.097 | .325 | *n.s.* |
|  | Group | .000 | .000, .027 | *F*(1,80) = 0.010 | .922 | *n.s.* |
| Correct_RT_SH | Time | .323 | --- | *F*(2,160) = 38.170 | .000 | *** |
|  | Interaction | --- | --- | *F*(166) = 0.122 | .835 | *n.s.* |
| *dL* | Group | .005 | .000, .072 | *F*(83) = 0.384 | .537 | *n.s.* |
|  | Time | .289 | --- | *F*(166) = 33.787 | .000 | *** |
|  | Interaction | .011 | --- | *F*(166) = 0.931 | .396 | *n.s.* |
| *CL* | Group | .001 | .000, .052 | *F*(83) = 0.097 | .756 | *n.s.* |
|  | Time | .014 | --- | *F*(166) = 1.147 | .320 | *n.s.* |
|  | Interaction | .009 | --- | *F*(166) = 0.741 | .478 | *n.s.* |
